# Traceless β-mercaptan-assisted activation of valinyl benzimidazolinones in peptide ligations†
†Electronic supplementary information (ESI) available. See DOI: 10.1039/c7sc04148a


**DOI:** 10.1039/c7sc04148a

**Published:** 2018-01-05

**Authors:** Yinglu Wang, Lin Han, Ning Yuan, Hanxuan Wang, Hongxing Li, Jinrong Liu, Huan Chen, Qiang Zhang, Suwei Dong

**Affiliations:** a State Key Laboratory of Natural and Biomimetic Drugs , Department of Chemical Biology , School of Pharmaceutical Sciences , Peking University , Beijing 100191 , China . Email: dongs@hsc.pku.edu.cn; b Department of Chemistry , University at Albany , Albany , New York 12222 , USA . Email: qzhang5@albany.edu

## Abstract

Peptidyl thioesters or their surrogates with C-terminal β-branched hydrophobic amino acid residues usually exhibit poor reactivities in ligation reactions. Thus, activation using exogenous additives is required to ensure an acceptable reaction efficiency. Herein, we report a traceless ligation at Val-Xaa sites under mild thiol additive-free reaction conditions, whereby the introduction of β-mercaptan on the C-terminal valine residue effectively activates the otherwise unreactive *N*-acyl-benzimidazolinone (Nbz), and enables the use of a one-pot ligation–desulfurization strategy to generate the desired peptide products. The orthogonality between β-thiovaline-Nbz and a conventional alkyl thioester, as well as the convenient access to the former from readily available penicillamine, also allowed expedited assembly of the peptidic hormone β-LPH and hPTH analogues, based on a kinetically controlled one-pot three-segment ligation and desulfurization strategy.

## Abstract 2

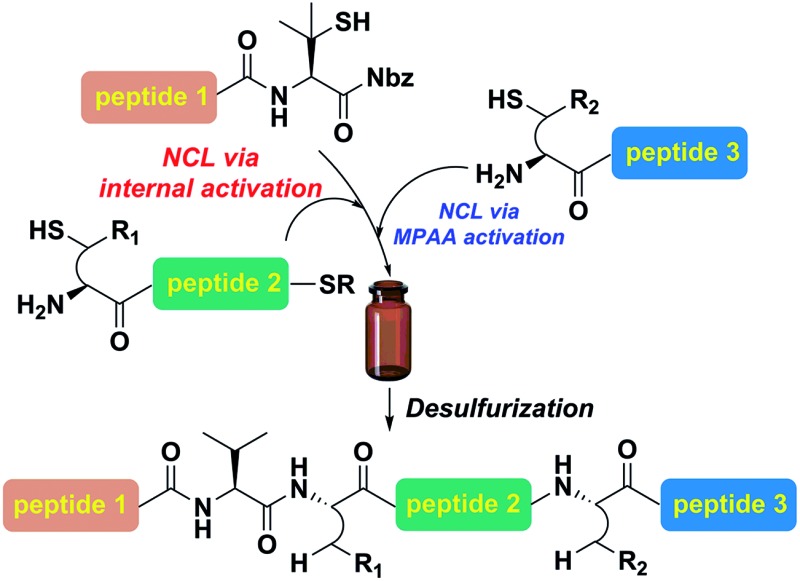
An internal activation strategy-enabled traceless ligation at sterically hindered Val-Xaa site is accomplished under thiol additive-free conditions assisted by a β-mercaptan on the C-terminal valine residue.

## Introduction

Native chemical ligation (NCL) is one of the most powerful and frequently used methods employed in the fields of chemistry and chemical biology^1^ for the construction of polypeptides and proteins.^2^ The versatility of the approach has encouraged continued development of surrogates, or “masked forms” of the required peptidyl thioesters,^3^ such as peptidyl hydrazide,^4^ peptide *N*-acyl-benzimidazolinone (peptide-Nbz),^5^ peptidyl selenoesters,^6^ and other amide-based precursors.^7^ Moreover, the logic of NCL has also inspired the desulfurization-enabled methodological expansion of optional peptide N-terminal residues in addition to the cysteines,^8^ along with other biocompatible ligation methods, including Staudinger ligation,^9^ α-ketoacid-hydroxylamine (KAHA) ligation,^10^ seleno-amino acid-based ligation,^11^ serine/threonine ligation (STL),^12^ and so on. While the ligation–desulfurization strategy has been widely applied in chemical protein synthesis, overcoming the detrimental steric effects involved in ligation has remained a challenge even with the assistance of exogenous thiol additives.^13^ Thus, innovative approaches to accelerate the reaction rate at bulky amino acid sites (*e.g.* Pro, Val, Ile, *etc.*) are desirable. Mild reaction conditions that are compatible with the broadly applied metal-free desulfurization (MFD) protocol,8*b* rather than those that utilize radical-quenching thiol additives such as 4-mercaptophenylacetic acid (MPAA),^14^would also be of benefit. Besides the search for more effective and practical additives,^15^ studies have also been conducted exploiting the presence of –SH functionalities within the peptide segments to activate the C-termini under thiol additive-free conditions,^16^ including a recent report from our group on a 4-thioproline-based ligation at the Pro-Xaa sites.^17^ In contrast to the internal activation originating from the cysteine residues within the sequence, a constrained thiolactone structure was suggested to be crucial in accounting for the drastically increased reactivity in comparison to the regular prolyl thioesters.^18^ Despite these improvements, methods that can improve efficiencies at other sterically demanding sites under mild reaction conditions are still of great importance, and call for further investigations.

**Fig. 1:**
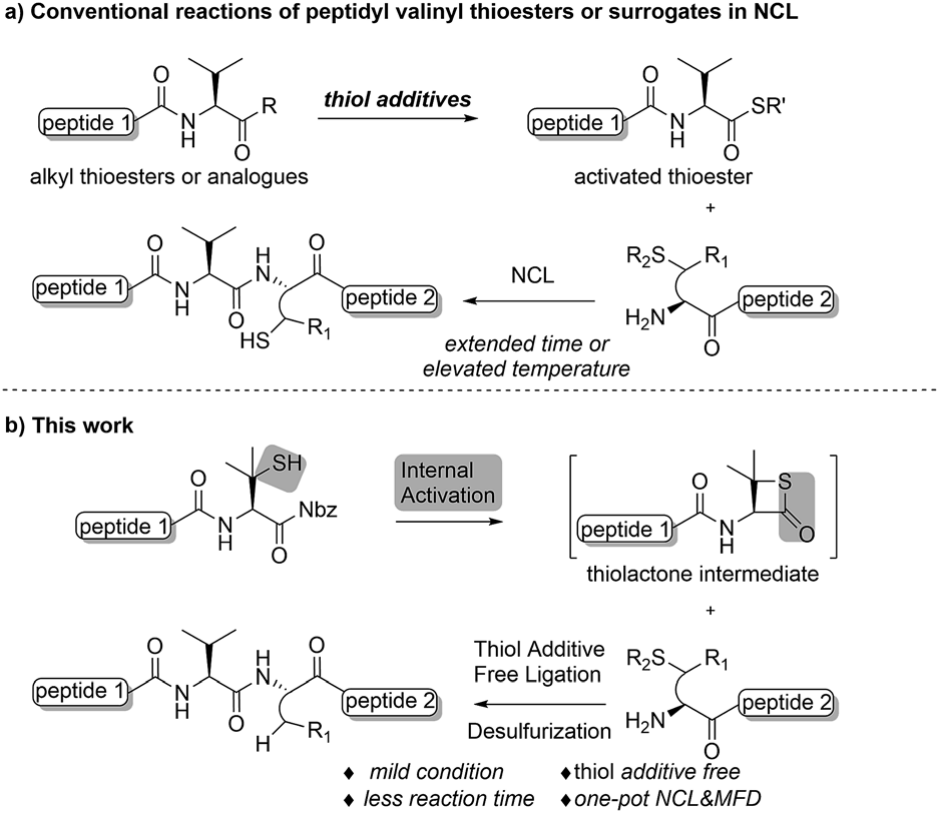
(a) Conventional reactions of peptidyl valinyl thioesters or surrogates *versus* (b) mercaptan-assisted internal activation of peptide valinyl-Nbz in NCL.

Despite being one of the most abundant amino acids in nature, connection sites in which valine is present are usually avoided in chemical ligations because of the sluggish reaction rate. Overcoming this constraint would afford more flexibility in choosing ligation sites for synthesizing intriguing peptides and proteins.^19^ Along this line, Seitz and co-workers have demonstrated that N-terminal penicillamine (Pen) could effectively mediate peptide ligation that eventually leads to an Xaa-Val linkage.^20^ While most of the developed methods accomplishing ligation at valine sites rely on a large excess of activating agents (Fig. 1a), we envisaged that the introduction of a C-terminal β-thiolactone moiety, possibly generated from a penicillamine-derived precursor *in situ*, might effect an increased reaction rate driven by the strain-releasing power of the reaction (Fig. 1b). To ensure the incorporation of the β-thio-valine structure in the peptides, the epimerization-free peptidyl-Nbz protocol developed by Dawson *et al.* could be applied, which would avoid extra modifications after SPPS.^5^

## Results and discussion

**Scheme 1:**
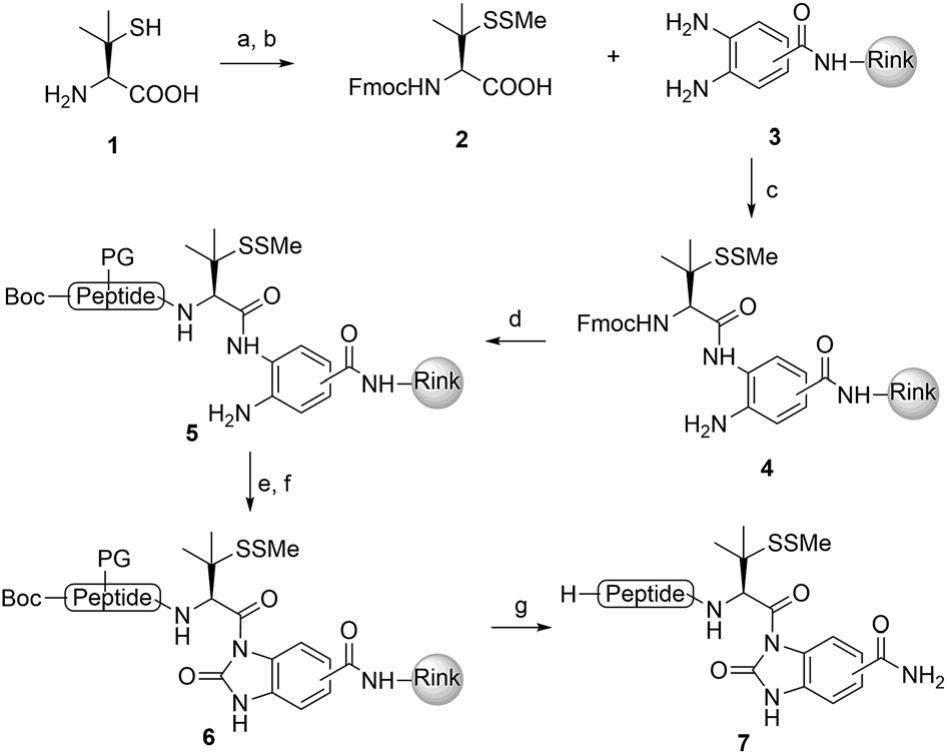
Synthesis of peptidyl-Nbz. Reagents and conditions: (a) MMTS, EtOH, rt, 4 h; (b) Fmoc-Cl, TEA, CH_2_Cl_2_, rt, overnight; (c) **3**, DIC, oxyma pure, DMF, 8 h; (d) Fmoc-SPPS; (e) *p*-nitrophenylchoroformate, DCM, 40 min to 1 h; (f) DIEA, DMF, 15 min; (g) TFA/TIS/H_2_O (95 : 2.5 : 2.5). DMF = *N*,*N*-dimethylformamide, DIEA = *N*,*N*-diisopropylethylamine, HATU = 1-[bis (dimethylamino)-methyl-ene]-1*H*-1,2,3-triazolo[4,5-*b*]pyridinium 3-oxid hexa-fluorophosphate, DBU = 1,8-diazabicyclo[5.4.0]undec-7-ene, DIC = *N*,*N*-diisopropylcarbodiimide, oxyma pure = ethyl cyanoglyoxylate-2-oxime, TFA = trifluoroacetic acid, TIS = triisopropylsilane, and PG = protecting groups.

We commenced our investigation by synthesizing the Pen-containing peptide-Nbz (Scheme 1). From readily available penicillamine (**1**), *N*-Fmoc protected amino acid derivative **2** was synthesized in two steps. It was subsequently installed on the 3,4-diaminobenzoic acid (Dbz)-preloaded Rink-amide resin **3** under DIC/oxyma pure conditions. The resulting resin was further employed in Fmoc-based SPPS, followed by the conversion of Dbz to Nbz based on literature-reported procedures.^5^ After the standard resin cleavage and global deprotections using a TFA cocktail, purification of the crude peptide using preparative reverse-phase HPLC provided the desired peptidyl-Nbz. Separately, the right-side peptides containing cysteines or other thio-amino acid derivatives at the N-termini were synthesized after the Fmoc-based SPPS and deprotection procedures.^21^

**Fig. 2:**
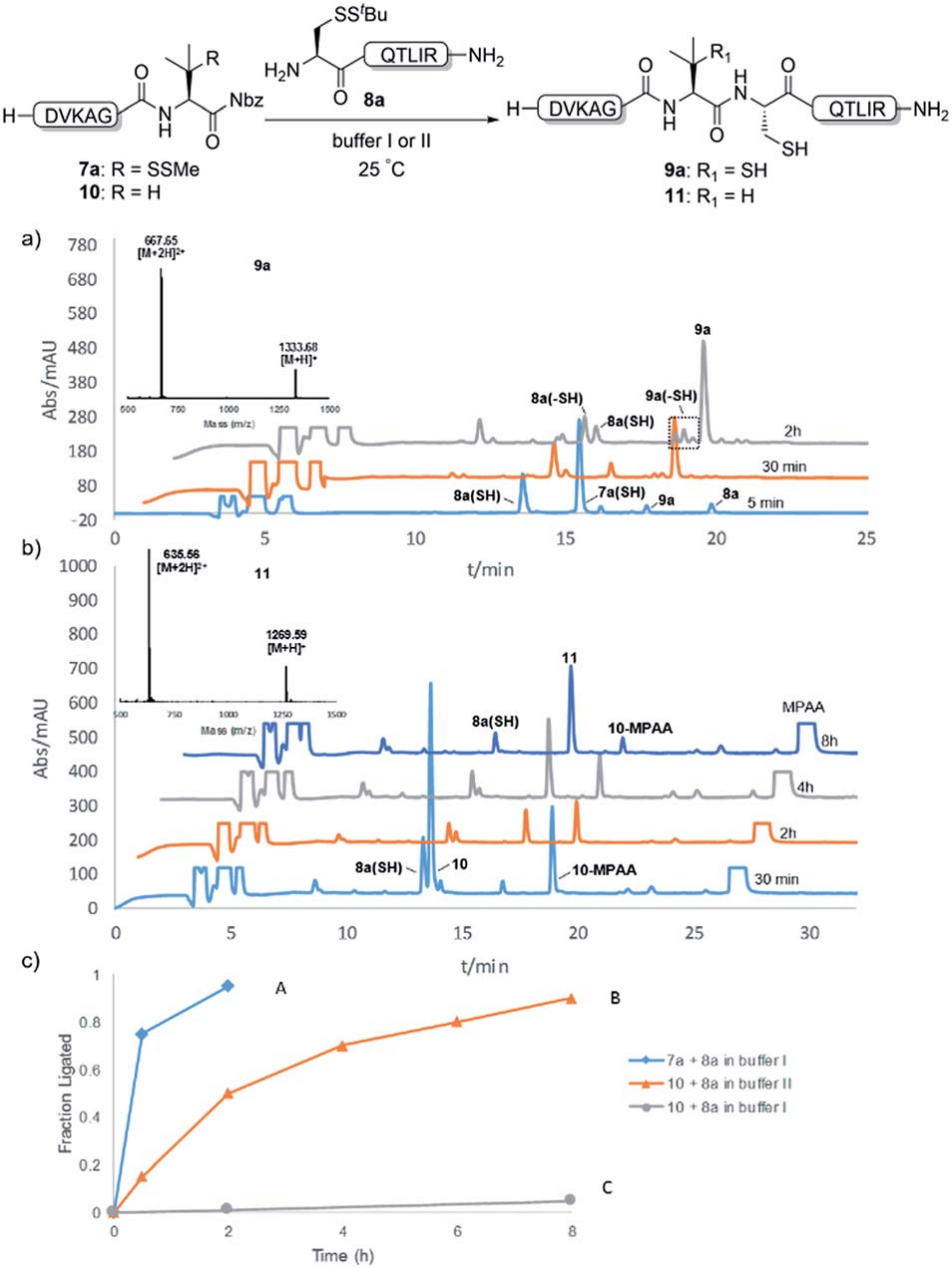
Evaluation of ligation efficiency using Pen or Val-containing peptide-Nbz in thiol additive-free buffer or MPAA-containing buffer. (a) UV trace of ligation between **7a** and **8a** in buffer I (6 M Gn·HCl, 200 mM Na_2_HPO_4_, 20 mM TCEP·HCl, pH 7.0); (b) UV trace of ligation between **10** and **8a** in buffer II (6 M Gn·HCl, 200 mM Na_2_HPO_4_, 200 mM MPAA, 20 mM TCEP·HCl, pH 7.0); (c) the reaction conversion as a function of time for the reactions between **7a**/**10** and **8a** in different buffers. Gn = guanidine and TCEP = tris(2-carboxyethyl) phosphine.

With the requisite peptide segments in hand, the ligation efficiency was evaluated using peptides H-DVKAGPen(SMe)-Nbz (**7a**, 1.0 equiv., Fig. 2) and H–C(S^*t*^Bu)QTLIR-NH_2_ (**8a**, 1.2 equiv.) under typical NCL reaction conditions at room temperature (6 M Gn·HCl, 200 mM Na_2_HPO_4_, 20 mM TCEP·HCl, pH 7.0). The reaction progress was monitored using HPLC-MS, and, as shown in Fig. 2a, peptidyl Pen-Nbz was efficiently transformed to product **9a** with high conversion after two hours, along with a trace amount of desulfurized products **8a** and **9a** (Fig. 2c, curve A). These desulfurized products may be derived from a TCEP-mediated reaction, where a phosphoranyl radical-based mechanism is possibly involved.8b,^22^ Similar observations have also been reported previously in the cases of ligation reactions in TCEP-containing buffer without any radical scavengers.^23^ Such side reactions could be suppressed by the addition of sodium ascorbate, which requires an extra HPLC purification before the metal-free desulfurization step,^16^,^23^ thus leading to decreased overall efficiency in comparison to the additive-free one-pot ligation–desulfurization protocol. In contrast, when peptide segment H-DVKAGV-Nbz (**10**), lacking β-thiol substitution at the C-terminal valine, was subjected to the same reaction conditions, a significantly lower reactivity was observed (Fig. 2c, curve C).^21^ The addition of MPAA accelerated the ligation of peptidyl Val-Nbz (Fig. 2b and c), which is in accordance with a previous report,^5^ but required a longer time than that of the β-thio Val-Nbz derived peptide to achieve over 90% conversion (*i.e.* 8 h). These results indicate that the β-mercaptan group on the terminal valine residue drastically increases the reaction rate.

**Fig. 3:**
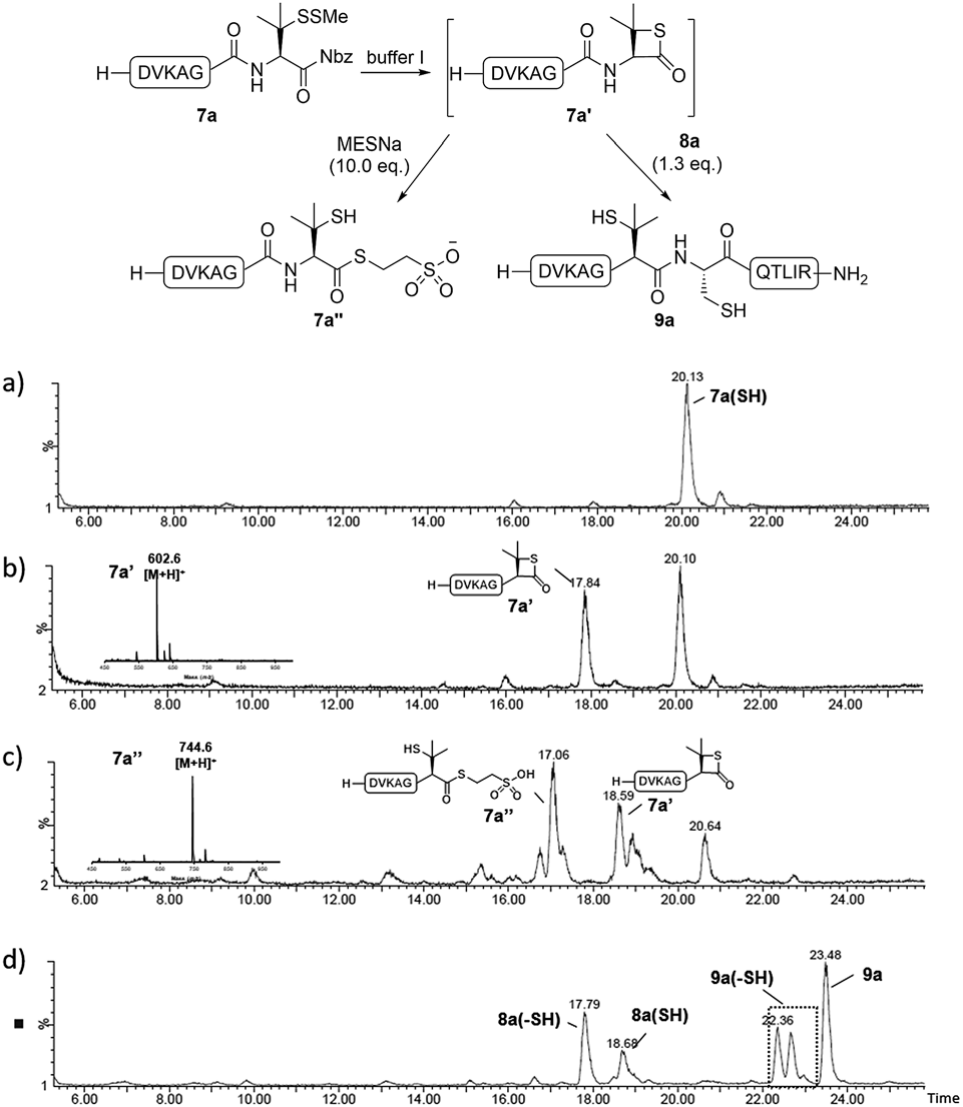
Mechanistic investigations. (a) Mass trace from the HPLC-MS analysis of **7a** dissolved in buffer I for 1 min; (b) mass trace of **7a** dissolved in buffer I for 30 min; (c) mass trace of the reaction between the mixture generated from stirring **7a** in buffer I for 30 min and 10 equiv. MESNa for 30 min; (d) mass trace of the reaction between the mixture generated from stirring **7a** in buffer I for 30 min and peptide **8a** for 2 h.

Since the proposed peptidyl thiolactone intermediate was not observed in the ligation between **7a** and **8a**, a series of control experiments were conducted to probe the penicillamine-Nbz-based reactions. When **7a** alone was dissolved in ligation buffer and monitored using HPLC-MS (Fig. 3), a new peak was observed after 30 min showing a mass corresponding to the β-thiolactone structure **7a′** (Fig. 3b), which could be either converted to alkyl thioester **7a′′** using excess MESNa (Fig. 3c), or intercepted by cysteinyl peptide **8a** to generate the ligated product **9a** (Fig. 3d). These results suggest the likely existence of the β-thiolactone structure, and rule out other possible intermediates such as lactams derived from intramolecular cyclizations.

Moreover, when two analogous peptides with either a terminal Cys-Nbz instead of Pen-Nbz (H-DVKAGC(S*^t^*Bu)-Nbz) or an internal Cys residue [H-DVKC(S^*t*^Bu)GV-Nbz] were subjected to the ligation reactions,^16^,^21^ an extremely low reactivity accompanied by extreme decomposition was observed (Fig. S24 and S25†). These findings further underscore the activating role played by the β-thiol at the C-terminal Val when compared to other internal activation methods using cysteines. Although our attempts to isolate the peptidyl thiolactone intermediate failed, due to severe decomposition during the HPLC purification and lyophilization of the sample, the experimental results suggest that the *gem*-dimethyl is crucial for the success of Pen-Nbz-based ligation under the reaction conditions, where the Thorpe-Ingold effect^24^ can stabilize the *in situ*-formed reactive four-membered-ring intermediate. Furthermore, the strain releasing power may accelerate the transthioesterification that is usually the rate-limiting step in cases of ligations at sterically hindered amino acid sites, accounting for the high reactivity observed. In another experiment, when N-terminal alanyl peptide H-AQTLIR-NH_2_ was subjected to ligation with **8a**,^21^ only a trace amount of the ligated product was generated (Fig. S26†), indicating that the reaction of Pen-Nbz likely proceeds through an NCL-like process instead of direct aminolysis.

In the absence of thiol additives, the ligation–desulfurization reaction of C-terminal Pen-Nbz-derived peptides can be conducted in a one-pot manner.15a,^25^ Several representative peptide-Nbz sequences containing different natural amino acids were subjected to the optimized conditions (Table 1), where the reactions with peptides containing *N*-cysteinyl peptides proceeded smoothly, affording the desired products in decent isolated yields over two steps (entries 1–3). Glycopeptide **8d** bearing an *N*-linked chitobiose was also found to be compatible under these conditions (entry 4). While the peptide-containing N-terminal β-thiol-Asp provided satisfactory reaction efficiency (entry 5),^26^ the ones with N-terminal thiol-valine derivatives displayed a much decreased reaction rate (entries 6 and 7),^20^,^27^ presumably due to the sterical constraints resulting from the consecutive Val–Val residues. It was notable that during our attempted desulfurization of thio-Asp-containing peptides using VA-044 as the radical initiator, significant amounts of side products were generated,25*b* which could be eliminated by using ACVA instead.^28^ Further attempts to react **7a** with N-terminal thio-proline-containing peptide failed to provide isolable ligation products,^21^ indicating that the activated penicillamine derivative was still unable to overcome the extreme steric effects between valine and proline.^29^ Moreover, a longer sequence, the 56 amino acid peptide γ-lipotropin, could also be efficiently prepared using this two-step one-pot protocol (entry 8).

**Table 1:** One-pot internal activated ligation–desulfurization of peptidyl Pen-Nbz^*a*^

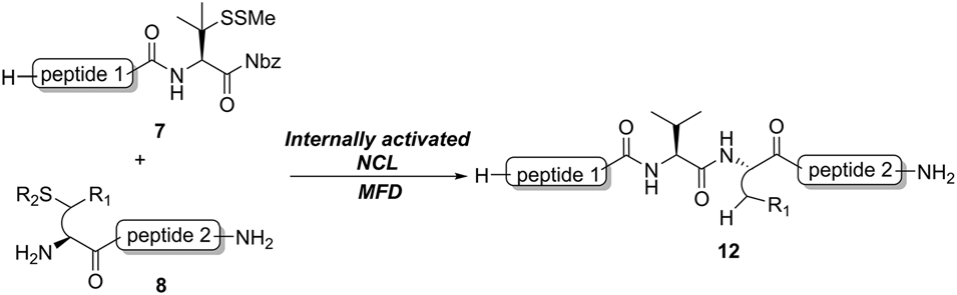			
Entry	Left-side peptide	Right-side peptide	Isolated yield (%)
1	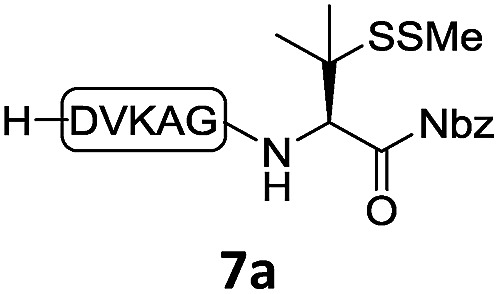	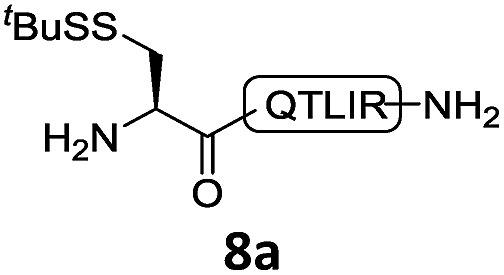	**12a**, 65%
2	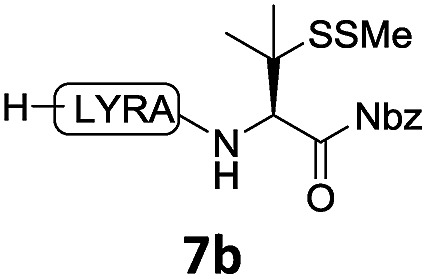	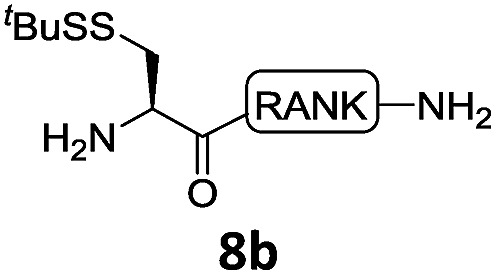	**12b**, 62%
3	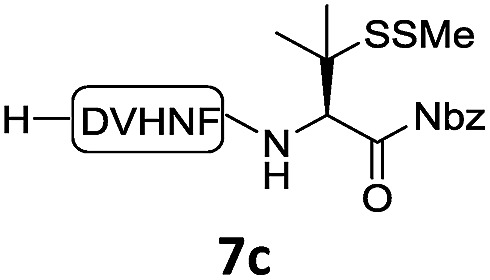	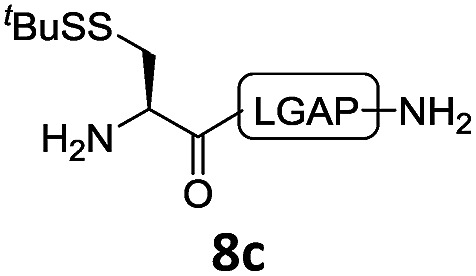	**12c**, 50%
4	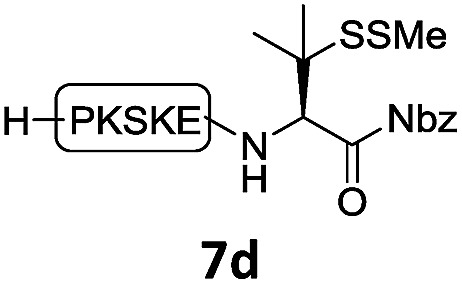	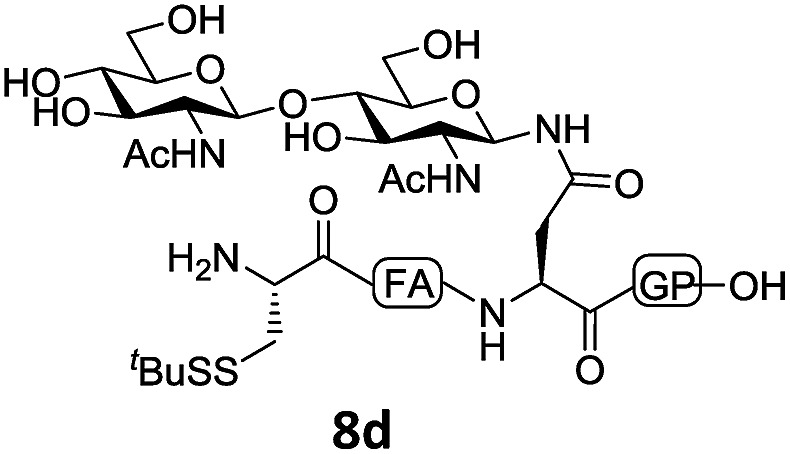	**12d**, 60%
5^*b*^	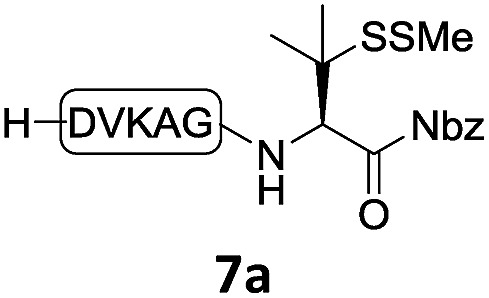	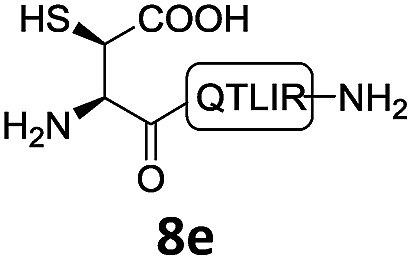	**12e**, 50%
6	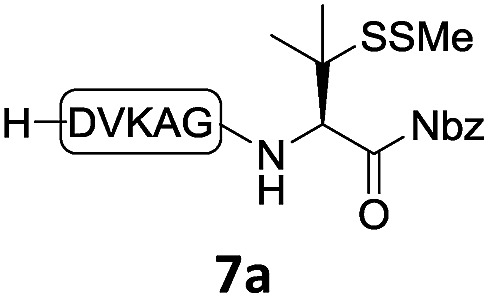	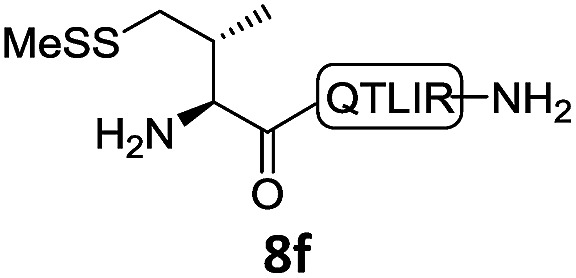	**12f**, 34%
7	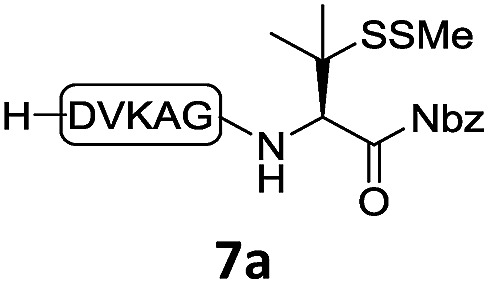	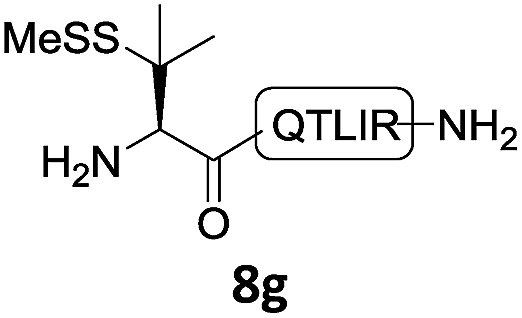	**12f**, 18%
8^*c*^	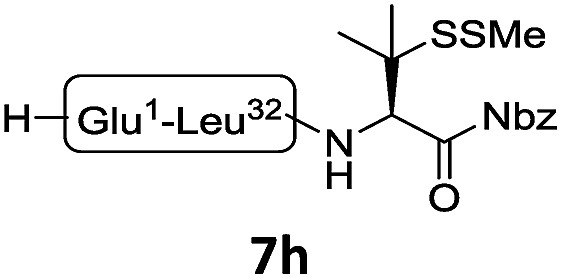	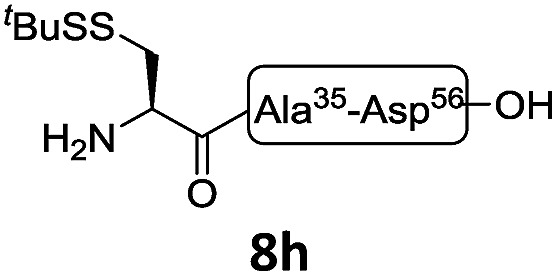	**12h**, 55%

^*a*^Reaction conditions: **7** (3 mM), 1.2 eq. **8**, NCL buffer (6 M Gn·HCl, 200 mM Na_2_HPO_4_, 20 mM TCEP·HCl, pH 7.0), 25 °C, 2 h, then bond-breaker (0.5 M TCEP), 20 μL ^*t*^BuSH, 100 μL VA-044 (0.1 M in H_2_O), 37 °C, 3 h.

^*b*^ACVA used in the desulfurization reaction instead of VA-044, 5 h.

^*c*^Reaction conditions: **7h** (5 mM), 1.2 eq. **8h**, 200 μL NCL buffer (6 M Gn·HCl, 200 mM Na_2_HPO_4_, 50 mM TCEP·HCl, pH 6.9), 25 °C, 8 h, then 200 μL bond-breaker (0.5 M TCEP), 20 μL ^*t*^BuSH, 100 μL VA-044 (0.1 M in H_2_O), 37 °C, 8 h. ACVA = 4,4′-Azobis(4-cyanovaleric acid) and VA-044 = 2,2′-Azobis[2-(2-imidazolin-2-yl) propane] dihydrochloride.

**Fig. 4:**
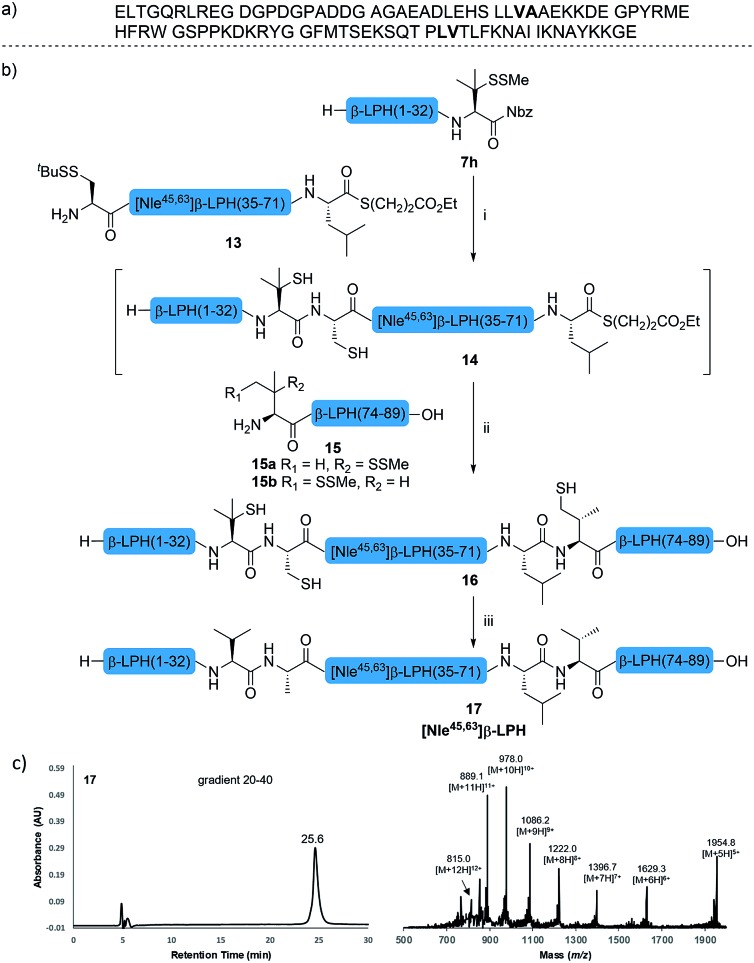
(a) Native sequence of β-LPH; (b) chemical synthesis of [Nle^45,63^] β-LPH. Reagents and conditions: (i) 6 M Gn·HCl, 100 mM Na_2_HPO_4_, 100 mM sodium ascorbate, 50 mM TCEP·HCl, pH 7.2, rt, 10 h; (ii) 6 M Gn·HCl, 200 mM Na_2_HPO_4_, 200 mM MPAA, 20 mM TCEP·HCl, rt, pH 7.0, 4 h. (iii) 6 M Gn·HCl, 200 mM Na_2_HPO_4_, pH 7.0, TCEP, VA-044, ^*t*^BuSH, 37 °C, 8 h, 31% yield over 3 steps. (c) HPLC trace and MS data of [Nle^45,63^] β-LPH.

**Fig. 5:**
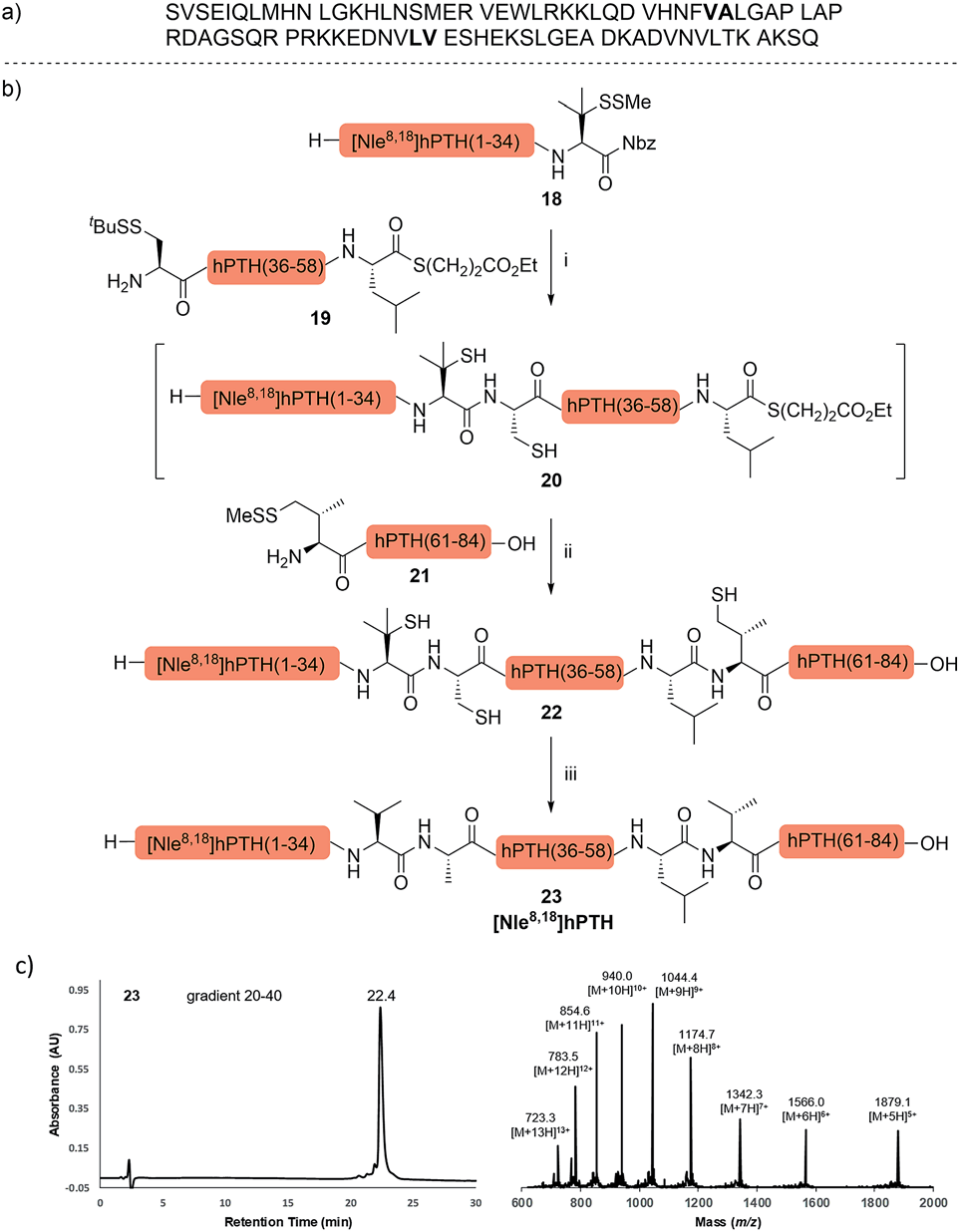
(a) Native sequence of hPTH; (b) chemical synthesis of [Nle^8,18^] hPTH. Reagents and conditions: (i) 6 M Gn·HCl, 100 mM Na_2_HPO_4_, 100 mM sodium ascorbate, 50 mM TCEP·HCl, pH 7.5, rt, 10 h; (ii) 6 M Gn·HCl, 200 mM Na_2_HPO_4_, 200 mM MPAA, 20 mM TCEP·HCl, rt, pH 7.2, 10 h. (iii) 6 M Gn·HCl, 200 mM Na_2_HPO_4_, pH 7.0, TCEP, VA-044, ^*t*^BuSH, 37 °C, 5 h, 35% yield over 3 steps. (c) HPLC trace and MS data of [Nle^8,18^] hPTH.

The internal thiol-assisted activation of Val-Nbz-derived peptides has also enabled the utilization of a kinetically controlled ligation (KCL) strategy in the synthesis of longer sequences,15a,^30^ where a peptidyl alkyl thioester would be unaffected in the thiol additive-free buffer, but could be activated later by an MPAA additive when needed. To validate the applicability of this strategy, we embarked upon the synthesis of β-lipotropin (β-LPH), a human peptidic hormone released from proopiomelanocortin (aka. POMC). β-LPH contains 89 amino acids (Fig. 4a), and can be endogenously cleaved to smaller peptidic hormones, including γ-LPH (Table 1, entry 8), β-MSH, and β-endorphin.^31^ Examination of the sequence revealed the absence of cysteines and the existence of two valine and two methionine residues, such that the full sequence could be assembled from three segments in a one-pot manner. The forward synthesis was first attempted by choosing Val^33^-Ala^34^ and Lys^54^-Asp^55^ as the ligation sites. To avoid possible oxidative degradation and enhance peptide stability, the methionines (Met^45^ and Met^63^) were replaced with norleucine (Nle) residues, an approach which has been suggested to mitigate oxidative degradation of peptides while maintaining their biological activity.^32^ Unfortunately, the product of the first ligation degraded rapidly in the MPAA buffer, and failed to connect with the third segment Lys^54^-Asp^55^. As we did not observe any lactamization or other obvious side products derived from the activated thioesters, the experimental result underscores the possible instability of peptide segments in the presence of a thiol additive under denaturing conditions, where more optional ligation methods and connection sites would be beneficial for identifying suitable synthetic routes leading to the desired target peptide.^33^ Alternatively, when the second ligation segment was extended to Leu^72^ (Fig. 4b), the corresponding ligation product **14** showed significantly improved stability, and was successfully ligated with peptide **15b** containing a γ-thiovaline derivative prepared following a palladium-catalyzed γ-C(sp^3^)–H acetoxylation protocol.^34^ It is notable that the N-terminal penicillamine-derived peptide **15a** was unreactive even at the elevated temperature of 40 °C. After the purification of full length β-LPH bearing three extraneous thiol groups using preparative HPLC to remove both sodium ascorbate and MPAA simultaneously, the subsequent desulfurization proceeded smoothly to generate [Nle^45,63^]β-LPH (**17**) in 31% yield over three steps and two HPLC purifications. In a similar manner, [Nle^8,18^]hPTH (**23**), an 84-*mer* protein analogue of human parathyroid hormone (hPTH, Fig. 5a), was successfully assembled with good efficiency and with fewer operational steps in comparison to the previous report,^35^ featuring a C-terminal β-thiovaline-Nbz ligation followed by an N-terminal γ-thiovaline ligation in the same flask (Fig. 5b). This further demonstrates the applicability of the KCL strategy enabled by the penicillamine-Nbz-derived peptides.

## Conclusions

In summary, we have developed a traceless ligation protocol at sterically demanding C-terminal valine sites, featuring an appropriately designed internal activation step mediated by a β-mercaptan substitution on the corresponding valine residue. In conjunction with the straightforward preparation of peptidyl penicillamine-Nbz segments, this effective thiol additive-free reaction ensured that the tandem desulfurization occurred in the same flask. This illustrates the compatibility of internal activation with alkyl thioesters, which is of practical use in the three-segment one-pot assembly of large polypeptides and proteins. Further studies on the mechanistic aspects of C-terminal penicillamine-based ligation, as well as the extension of the strain-releasing activation strategy in peptide ligation at other difficult sites, are currently in progress and will be reported in due course.

## Conflicts of interest

The authors declare no competing financial interest.

## Supplementary Material

Supplementary information
