# Supplementary material for: Traceless β-mercaptan-assisted activation of valinyl benzimidazolinones in peptide ligations
Source: Chem Sci. 2018 Jan 5;9(7):1940–6. doi: 10.1039/c7sc04148a (PMC5892131; doi:10.1039/c7sc04148a)
Supplement: Supplementary file 1 [file SC-009-C7SC04148A-s001.pdf]

Electronic Supplementary Material (ESI) for Chemical Science.
This journal is © The Royal Society of Chemistry 2018

Supporting Information

Traceless β-Mercaptan-Assisted Activation of Valinyl Benzimidazolinones in Peptide Ligations

Yinglu Wang,[†] Lin Han,[†] Ning Yuan,[†] Hanxuan Wang,[†] Hongxing Li,[†] Jinrong Liu,[†] Huan Chen,[‡] Qiang Zhang*,[‡] and Suwei Dong*,[†]

[†]*State Key Laboratory of Natural and Biomimetic Drugs, and Department of Chemical Biology, School of Pharmaceutical Sciences, Peking University, 38 Xueyuan Road, Haidian District, Beijing 100191, China*

E-mail: dongs@hsc.pku.edu.cn

[‡]*Department of Chemistry, University at Albany, 1400 Washington Avenue CH 122, Albany, NY 12222, United States*

E-mail: qzhang5@albany.edu

Table of Contents

I. General Information....................S3

II. Synthesis and Characterization of Penicillamine Derivative....................S5

III. General Procedures for Peptide Synthesis S10

IV. Preparation and Characterization of Peptide Segments....................S14

V. Ligation Reactions Probing the Mechanistic Insight....................S31

VI. One-Pot Ligation and Desulfurization Reactions S33

VII. Synthesis of [Nle^{45,63}]β-LPH (17) and [Nle^{8,18}]hPTH (23)....................S42

I. General Information

Materials and Methods

All commercial materials (Sigma Aldrich, Fisher, Acros, TCI, Adamas, J&K, GL Biochem, Energy, *etc*.) were used without further purification. All solvents were reagent grade or HPLC grade (Fisher, Sigma, Acros, Oceanpak). Anhydrous tetrahydrofuran, dichloromethane, diethyl ether, toluene, and *N,N*-dimethyl formamide were purified and dried by PURE SOLV® solvent purification system (Innovative Technology, Inc.). Analytical thin layer chromatography was performed using Merck TLC silica gel 60-F254 glass plates. Flash chromatography was performed using 200-300 mesh silica gel (Qingdao Haiyang Chemical Co., Ltd.). Filtration for crude peptide was performed using a Bulk GHP Acrodisc® 13 mm syringe filter with 0.22 μm GHP membrane. Ultra-pure argon (⩾99.999%) was used in all ligation and desulfurization reactions. Yields refer to chromatographically and spectroscopically pure materials unless otherwise stated.

^{1}H NMR spectra were recorded on Bruker Avance III 400 MHz at ambient temperature using $CDCl_3$ as solvent unless otherwise stated, referenced to TMS or residual solvent. ^{13}C NMR spectra were recorded at 100.0 MHz at ambient temperature using $CDCl_3$ as solvent unless otherwise stated. Chemical shifts are reported in parts per million relative to $CDCl_3$(^{1}H, δ 7.26; ^{13}C, δ 77.0). Data for ^{1}H NMR are reported as follows: chemical shift, integration, multiplicity (ovrlp = overlapping, s = singlet, d = doublet, t = triplet, q = quartet, m = multiplet) and coupling constants (*J* Hz). All ^{13}C NMR spectra were recorded as chemical shift (δ). Infrared spectra were recorded on a Nicolet Nexus 670 FT-IR spectrophotometer. High-resolution mass spectra were obtained in the Chemical Instrumentation Center, Peking University Health Science Center using a Waters Q-TOF mass spectrometer (Xevo G2 Q-TOF). Low-resolution mass spectra analyses were performed with a Waters SQD mass spectrometer (Alliance e2695-SQD). Melting points were recorded on a melting point-M560 (Buchi). Optical rotations were recorded on an AUTOPOL III digital polarimeter at 589 nm and are recorded as $[\alpha]_D^{22}$

(concentration in grams/100 mL solvent).

All HPLC separations involved a mobile phase of 0.05% (*v/v*) TFA in water (solvent A) and 0.04% (*v/v*) TFA in acetonitrile (solvent B).

Analytical LC-MS analyses were performed using a Waters Alliance e2695 Separations Module and a Waters 2489 UV/Visible (UV/Vis) Detector equipped with an Agilent C18 column (5.0 μm, 4.6 × 150 mm) at a flow rate of 0.4 mL/min, a Beim Brueckle C4 column (5.0 μm, 4.6 × 150 mm) at a flow rate of 0.4 mL/min, or a Higgins Analytical PROTO-300 C4 (5.0 μm, 2.1 × 150 mm) at a flow rate of 0.2 mL/min. The wavelengths of UV-detector were set to 210 nm and 220 nm.

Analytical HPLC separations were performed using an Agilent Technologies 1260 Infinity LC system.

Preparative HPLC separations were performed using a Hanbon Sci. & Tech. NP7005C solvent delivery system and a Hanbon Sci. & Tech. NU3010C UV detector equipped with an Agilent Eclipse XDB-C18 column (7.0 μm, 21.2 × 250 mm), a Beim Brueckle C4 column (10.0 μm, 20 × 250 mm), or a Proto 300 C4 column (10.0 μm, 20 × 250 mm) at a flow rate of 16 mL/min. The wavelengths of UV-detector were set to 210 nm and 220 nm.

II. Synthesis and Characterization of Penicillamine Derivative

Synthesis of the N-9-fluorenylmethoxycarbonyl-methylthio-L-penicillamine (2)

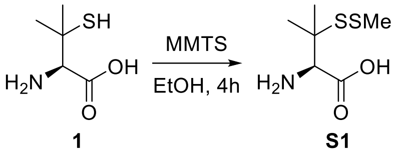

To a solution of penicillamine (300 mg, 2.0 mmol) in 15 mL of degassed EtOH was added methyl methanethiosulfonate (MMTS, 0.2 mL, 2.0 mmol) dropwise. The mixture was stirred at room temperature for 4 h. Upon the full consumption of penicillamine, the reaction was concentrated in vacuo to afford compound **S1** without further purification.

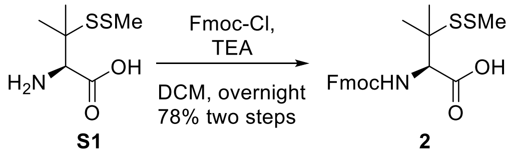

To a solution of crude **S1** and 9-fluorenylmethyl chloroformate (Fmoc-Cl, 624 mg, 2.4 mmol) in 15mL DCM, triethylamine (TEA, 0.67 mL, 4.8 mmol) was added dropwise at 0 °C. The reaction was stirred at room temperature under an argon atmosphere overnight, then quenched with 1 M aqueous HCl at 0 °C. The mixture was extracted three times with EtOAc, and the combined extracts were washed with H_2O and brine, dried over magnesium sulfate, and concentrated in vacuo. The crude residue was purified using silica gel column chromatography eluting with petroleum ether/EtOAc (5:1) containing 1% of HOAc to afford compound **2** as a white solid (656 mg, 78% two steps). IR (thin film): ν_{max} 2970, 1719, 1513, 1450, 1417, 1368, 1340, 1226, 1152, 1119, 1048, 1008, 912 cm^{-1}; $[\alpha]^{20}_{D}$ = + 9.7° (*c* 0.01, $CHCl_3$); m.p. 74-75 °C; ^1H NMR (400 MHz, $CDCl_3$): δ 7.76 (d, *J* = 7.4 Hz, 2H), 7.64-7.57 (m, 2H), 7.44-7.36 (m, 2H), 7.36-7.28 (m, 2H), 5.64 (d, *J* = 8.8 Hz, 1H), 4.57-4.30 (m, 3H), 4.24 (t, *J* = 6.8 Hz, 1H), 2.40 (s, 3H), 1.53-1.31 (m, 6H); ^{13}C NMR (100 MHz, $CDCl_3$): δ 174.9, 156.0, 143.7, 143.6, 141.3 (two carbons overlap), 127.7 (two carbons overlap), 127.1 (two carbons overlap), 125.1 (two carbons overlap), 120.0 (two carbons overlap), 67.3, 60.9, 52.1, 47.1, 26.3, 25.1, 24.5; HRMS-ESI (*m/z*): calcd for $C_{21}H_{23}NO_2S_2$ $[M+H]^+$: 418.1141, $[M+Na]^+$:

440.0961; found: 418.1141, 440.0959.

Synthesis of (2S, 3R)-2-(tert-Butoxycarbonylamino)-4-(2-tret-Butyldisulfulfanyl)- L-valine

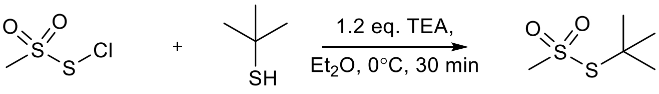

S2

Compound **S2** was synthesized following literature reported procedures.[S1] To the solution of methanesulfonyl chloride (77 µL, 1.0 equiv) and 2-methyl-2-propanethiol (0.11 mL, 1.0 equiv) in Et_2O (1.0 mL) was added TEA (0.14 mL, 1.2 equiv) dropwise at 0 ˚C. The reaction was stirred at 0 ˚C for 30 min and then quenched with 1N HCl and water at 0 ˚C. The resulting mixture was extracted with ethyl acetate (×3), and the combined extracts were washed with water and brine, dried over $MgSO_4$ and concentrated in vacuo. The crude material was purified using silica gel column chromatography (petroleum ether: CH_2Cl_2 = 5:1) to afford compound **S2** as a colorless oil (150 mg, 90%).

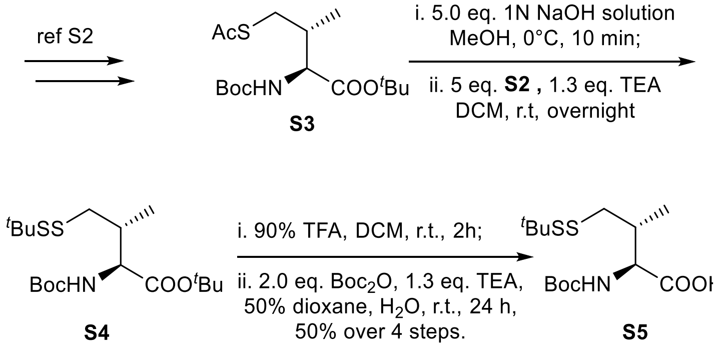

A solution of compound **S3**[S2] (160 mg, 1.0 equiv) in methanol (3.7 mL) was treated with 1N NaOH solution (5.5 ml, 5.0 equiv) at 0˚C for 10 min. The reaction mixture was carefully neutralized using 1N HCl and water at 0˚C. The resulting mixture was extracted with ethyl acetate (×3). The combined extracts were washed with water and brine, dried with $MgSO_4$ and concentrated in vacuo. The obtained crude intermediate was used directly in next step without purification, which was mixed with **S2** (270 mg,

---

[S1] H. T. Pham, N.-L. T. Nguyen, F. Duus, T. X. T. Luu, *Phosphorus, Sulfur, and Silicon and the Related Elements* **2015**, *190*, 1934-1941.
[S2] K. K. Pasunooti, R. Yang, B. Banerjee, T. Yap, C. F. Liu, *Org. Lett.* **2016**, *18*, 2696-2699.

3.5 equiv) in anhydrous CH_2Cl_2 (3 mL), followed by the addition of TEA (80 μL, 1.2 equiv) at 0 ˚C. The reaction mixture was stirred at room temperature overnight, then quenched with 1N HCl and water at 0˚C. The resulting mixture was extracted with ethyl acetate (×3). The combined extracts were washed with water and brine, dried with $MgSO_4$ and concentrated in vacuo to afford crude compound **S4**, which was treated with TFA/DCM = 9:1 (*v/v*) solution at room temperature for 2 hours. The solvent was removed under a nitrogen atmosphere. The residue was co-evaporated with toluene, and dissolved in 3 mL dioxane/H_2O (1:1, *v/v*), followed by the addition of Boc_2O (216 μL, 2.0 equiv) and TEA (80 μL, 1.3 equiv) at 0 ˚C. After stirring at room temperature for 24 hours, the reaction was quenched with 1N HCl and water at 0 ˚C. The resulting mixture was extracted with ethyl acetate (×3). The combined extracts were washed with water and brine, dried with $MgSO_4$ and concentrated in vacuo. The crude residue was purified using silica gel column chromatography eluting with petroleum ether/EtOAc (6:1) containing 1% of HOAc to afford compound **S5** as a light yellow syrup (77 mg, 50% in 4 steps). IR (thin film): ν_{max} 2969, 2931, 1716, 1506, 1455, 137, 1164, 1058 cm^{-1}; $[\alpha]^{24.9}_{D}$ = - 63.2˚ (*c* 1.0, MeOH); ^1H NMR (400 MHz, $CDCl_3$): δ 5.13 (d, *J*=7.6 Hz 1H), 4.43-4.26 (m, 1H), 2.98-2.85 (m, 1H), 2.65-2.47 (m, 1H), 2.41-2.28 (m, 1H), 1.45 (s, 9H), 1.32 (s, 9H), 1.09 (d, *J*=6.3 Hz, 3H); ^{13}C NMR (100 MHz, $CDCl_3$): δ 176.1, 155.6, 80.3, 57.3, 47.9, 43.8, 36.5, 29.9 (three carbons overlap), 28.3 (three carbons overlap),15.6; HRMS-ESI (*m/z*): calcd for $C_{14}H_{26}NO_4S_2$ $[M-H]^-$: 336.1303; found, 336.1297.

NMR Spectra. of amino acids derivatives

Compound 2 - ^{1}H NMR Spectrum - $CDCl_3$, 400 MHz

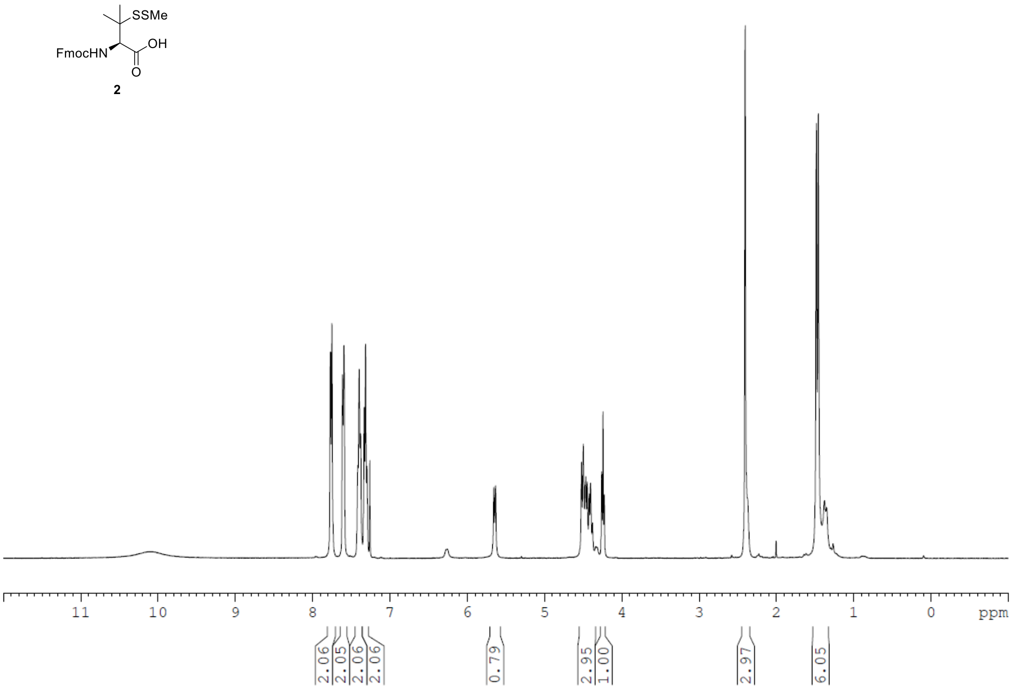

Compound 2 – ^{13}C NMR Spectrum - $CDCl_3$, 100 MHz

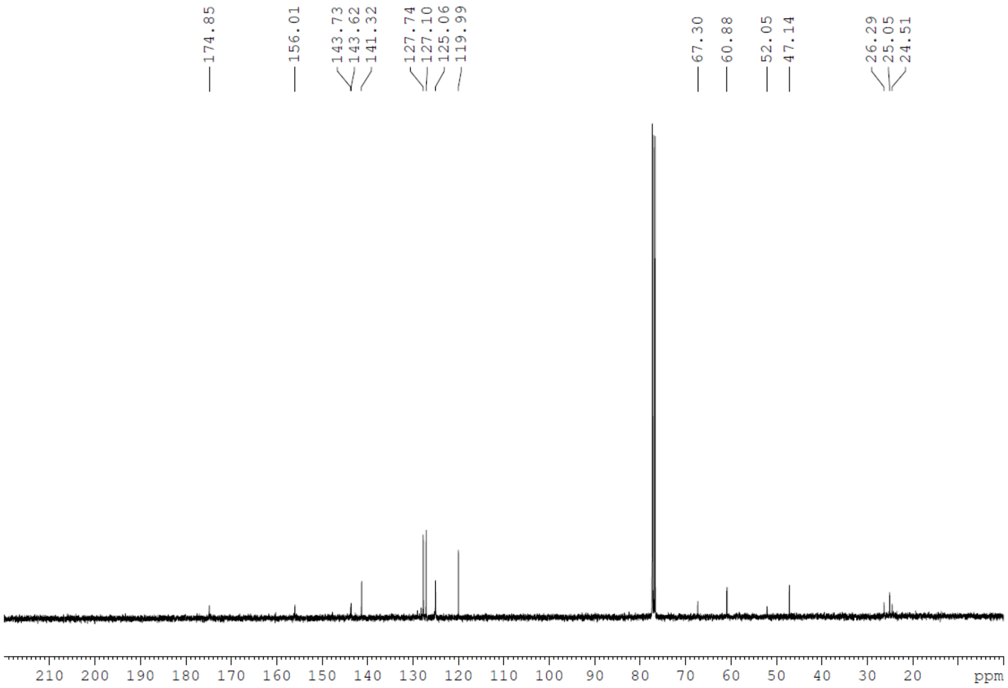

Compound S5 - ^{1}H NMR Spectrum - $CDCl_3$, 400 MHz

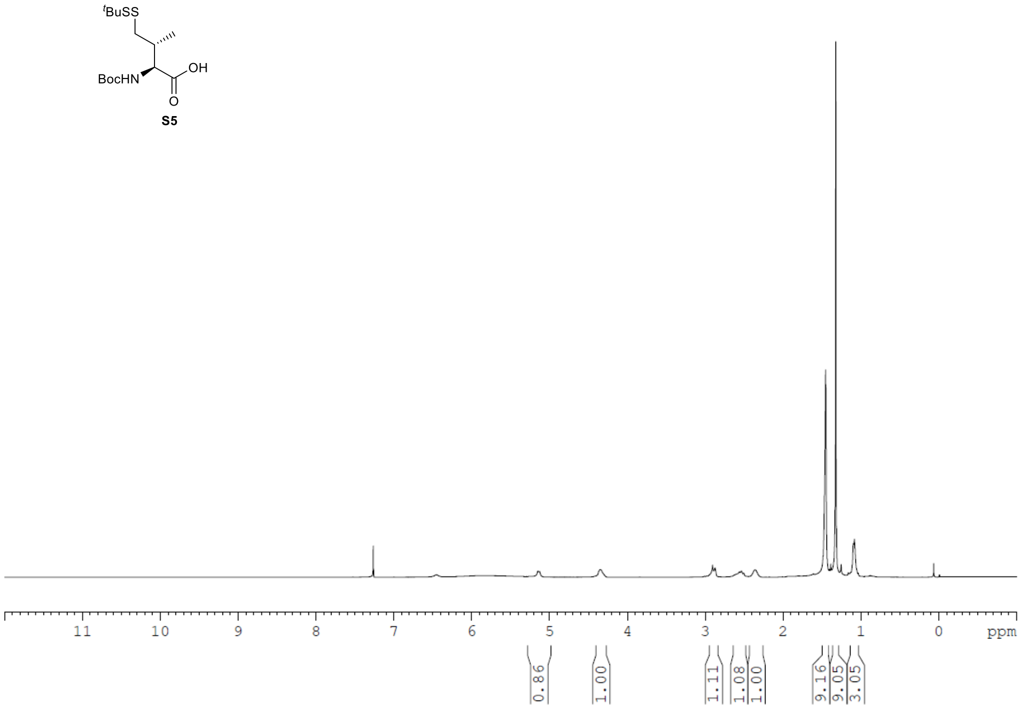

Compound S5 – ^{13}C NMR Spectrum - $CDCl_3$, 100 MHz

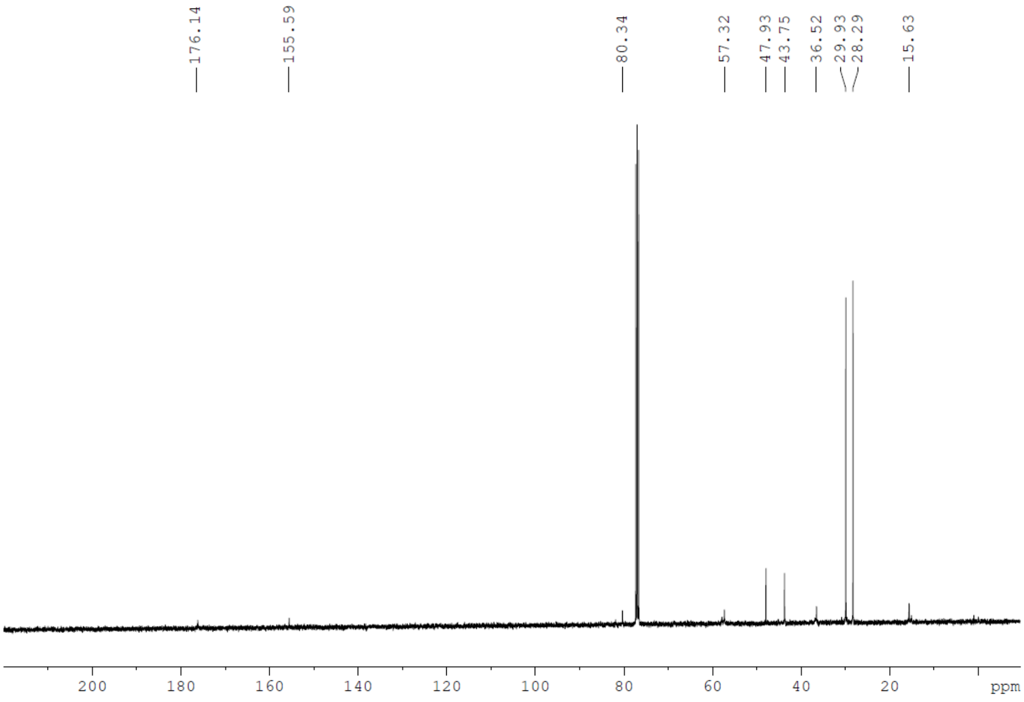

III. General Procedures for Peptide Synthesis

3.1 Preparation of amino acid pre-loaded resin and determination of resin loading

Pre-load an amino acid to 2-chlorotritylchloride resin

The first Fmoc-amino acid residue was loaded to 2-chlorotritylchloride resin before Fmoc-SPPS following the general procedure below.

To a mixture of Fmoc-amino acid (1.0 equiv) and 2-chlorotritylchloride resin was added dry DCM (approx. 10 mL per gram of resin) and DIEA (4.0 equiv). The reaction was agitated for 2 hours. The resin was collected and washed with 17/2/1 (*v*/*v*/*v*) of DCM/MeOH/DIEA (×3), DCM (×3), DMF (×2), DCM (×3), and dried in vacuo for 12 hours before the loading test.

Pre-load Fmoc-Dbz to Rink-MBHA resin

The Rink-MBHA resin (1.0 equiv) was deprotected with 2% DBU and 2% piperidine in DMF for 5 min (×2) to remove the Fmoc group. The synthesized Fmoc-Dbz (4.0 equiv),[S3] HATU (4.0 equiv) in DMF (approx. 10 mL per gram of resin) was added to the resin. DIEA (8.0 equiv) was added dropwise to the reaction mixture. The reaction was agitated for 1 hour. The resin was collected and washed with DMF (×2), DCM (×3), DMF (×2), DCM (×3), and dried in vacuo for 24 hours before the loading test.

Determination of resin loading[S4]

Dry Fmoc amino-acid resin (approx. 5 μmol with respect to Fmoc) was weighted into a clean test tube, followed by the addition of 2 mL of 2% DBU in DMF. The mixture was agitated gently for 30 min, and then diluted to 10 mL with CH_3CN. 2 mL of the resulting solution was taken out and diluted to 25 mL in a 50 mL centrifuge tube as the test solution. A reference solution was prepared in the same manner without the addition of resin.

Two matched silica UV cells were filled with reference solution to blank the U.V. spectrophotometer. The solution in one of the silica UV cells was changed to the test

[S3] J. B. Blanco-Canosa, P. E. Dawson, *Angew. Chem. Int. Ed.* **2008**, *47*, 6851-6855.
[S4] Peptide Synthesis, 2010/2011 Catalog, Merck.

solution after washing with the test solution for three times. The optical density at 304 nm was recorded for three times and the average value was calculated as Abs_{sample}. The Fmoc loading of resin could be calculated using the equation below:

$$\text{Fmoc loading: mmol/g} = Abs_{sample} \times 16.4/(\text{mg of resin}).$$

3.2 Automated solid-phase peptide synthesis

Automated peptide synthesis was performed on a Pioneer peptide synthesis system (GEN600611) or a CS Bio peptide synthesizer (CX136XT).

Pioneer peptide synthesizer Peptides were synthesized under standard automated Fmoc protocols using DMF as solvent, deblocking for 5 min in piperidine/DBU/DMF (2:2:96, *v/v/v*), coupling for 25 min ('standard cycle'), or 55 min ('extended cycle') for amino acids after steric hindered residues such as prolines, valines, threonines, isoleucines and arginines using HATU as coupling reagent.

CS Bio peptide synthesizer Peptide synthesis was performed following the general protocol using DMF as solvent, deblocking (5 min × 2) in piperidine/DMF (20:80, *v/v*) containing Oxyma (0.1 M), couple for 25 min using HATU/HOBt (1:1) as coupling reagent, for amino acids after steric hindered residues, the coupling cycle was repeated as needed.

The following $^{\alpha}N$-Fmoc or $^{\alpha}N$-Boc-protected amino acids and pseudoproline dipeptides from Novabiochem, GL Biochem or CS Bio were employed in SPPS: Fmoc-Ala-OH, Fmoc-Arg(Pbf)-OH, Fmoc-Asn(Trt)-OH, Fmoc-Asp(O^{t}Bu)-OH, Fmoc-Glu(O^{t}Bu)-OH, Fmoc-Gln(Trt)-OH, Fmoc-Gly-OH, Fmoc-His(Trt)-OH, Fmoc-Ile-OH, Fmoc-Leu-OH, Fmoc-Lys(Boc)-OH, Fmoc-Met-OH, Fmoc-Phe-OH, Fmoc-Pro-OH, Fmoc-Ser(^{t}Bu)-OH, Fmoc-Thr(^{t}Bu)-OH, Fmoc-Trp(Boc)-OH, Fmoc-Tyr-OH, Fmoc-Val-OH, Fmoc-Nle-OH, Fmoc-Asn(Trt)-Ser($\psi^{Me,Me}$Pro)-OH, Boc-Glu(O^{t}Bu)-OH, Boc-Cys(S^{t}Bu)-OH, Boc-Cys(Trt)-OH, Boc-Ser(^{t}Bu)-OH.

The employed 2-chlorotritylchloride resin (1.147 mmol/g) employed in SPPS was purchased from GL Biochem, and Rink MBHA resin (0.42 mmol/g) was purchased from CS Bio.

3.3 Preparation of peptidyl acids and peptidyl amides

Upon completion of the automated synthesis on a 0.05 mmol scale, the peptide resin was washed into a peptide synthesis vessel using DCM. Resin cleavage and global deprotection was performed under the treatment of TFA/H_2O/TIPS (95:2.5:2.5, *v/v/v*) solution for 2 hours. The resin was then removed by filtration, and the filtrate was concentrated under a nitrogen atmosphere. The residue was washed with cold diethyl ether to give a white solid, which was then dissolved in a mixture of acetonitrile and water containing 5% of acetic acid. The resulting solution was ready for HPLC purification after filtration. Utilization of 2-chlorotrityl resin and Rink- MBHA resin afforded the peptidyl acids and peptidyl amides respectively.

For side-chain protected peptide, the resin after SPPS was treated with DCM/TFE/AcOH (3:1:1, *v/v/v*) solution 40 min (×3), the combined cleavage solution was concentrated under reduced pressure, and lyophilized to remove the residual acid.

3.4 Preparation of *C*-terminal Pen-Nbz containing peptides

Fmoc-Pen(SMe)-OH (**2**) (5.0 equiv) was coupled to pre-loaded Dbz-Rink-MBHA resin (1.0 equiv, loading = 0.307 g/mol) manually using DIC (5.0 equiv) and oxyma pure (5.0 eq.) in DMF for 8 hours. The resulting resin was washed with DMF (×2), DCM (×3), DMF (×2) and DCM (×3), followed by the automated synthesis on a 0.02-0.05 mmol scale, where the last residue was introduced as Boc-Xaa.

After peptide elongation, the resin was washed with DCM and treated with *p*-nitrophenylchloroformate solution (50 mM in dry DCM) for 40 min at room temperature, unless otherwise stated. Then it was washed with DCM (×3), DMF (×2), treated with DIEA (0.5 M in DMF) for 15 min to convert Dbz to Nbz.[S3] Finally, the resin was washed thoroughly using DMF/DCM. Resin cleavage and global deprotection was performed under the treatment of TFA/H_2O/TIPS (95:2.5:2.5, *v/v/v*) solution for 2 hours. The resin was then removed by filtration, and the filtrate was concentrated under a nitrogen atmosphere. The residue was washed with cold diethyl ether to give a white solid, which was then dissolved in a mixture of acetonitrile and water containing 5% of acetic acid. The resulting solution was ready for HPLC

purification after filtration.

3.5 Native Chemical Ligation

To a mixture of the west-side peptide (1.0 equiv), and east-side peptide containing thiol-amino acid at the *N*-terminus (1.2 equiv), was added appropriate volume of ligation buffer (6 M Gn·HCl, 200 mM Na_2HPO_4, 20 mM TCEP·HCl, pH 7.0) under an argon atmosphere, the concentration of the west-side peptides is approximately 3 mM, and the resulting solution was stirred at room temperature (25 °C) and monitored using LC-MS. The reaction was quenched with H_2O/MeCN/AcOH (90:5:5, *v/v/v*) and purified using HPLC.

3.6 Metal-free Desulfurization

To a solution of the thiol-containing peptide (3 mM) in appropriate volume of degassed buffer (6 M Gn•HCl, 200 mM Na_2HPO_4, pH 7.0) was added 0.5 M Bond-breaker® TCEP solution (Pierce), 20 μL of 2-methyl-2-propanethiol and 100 μL of radical initiator VA-044 (0.1 M in degassed water). The reaction mixture was stirred at 37 °C and monitored by LC-MS. Upon completion, the reaction was quenched by adding H_2O/MeCN/AcOH (90:5:5, *v/v/v*) and further purified using HPLC.

3.7 One-pot Ligation-Desulfurization

Upon completion of the ligation as indicated by LC-MS analysis, to the reaction vessel was added proper volume of 0.5 M Bond-breaker® TCEP solution (Pierce), 20 μL of 2-methyl-2-propanethiol and 100 μL of radical initiator VA-044 (0.1 M in degassed water). The reaction mixture was stirred at 37 °C and monitored by LC-MS. Upon completion, the reaction was quenched by adding H_2O/MeCN/ AcOH (90:5:5, *v/v/v*) and further purified using HPLC.

IV. Preparation and Characterization of Peptide Segments

Peptidyl Pen-Nbz 7a

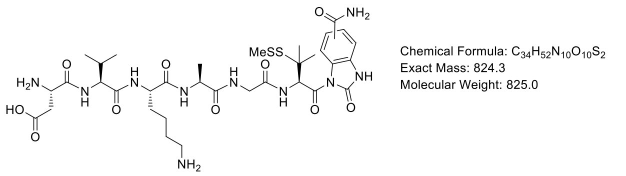

H-DVKAGPen-Nbz (7a)

Peptide **7a** (mixture of 3-/4-substitued Nbz isomers) was prepared according to General Procedure **3.4** using Pioneer peptide synthesizer on a 0.03 mmol scale. Purification of the crude peptide using preparative HPLC (10 to 30% solvent B over 30min, Agilent Eclipse XDB-C18 column) afforded peptide **7a** as a white solid after lyophilization (11.1 mg, 45%). Analytical HPLC: t_R = 18.1, 18.8 min (10 to 40% solvent B over 30min, Agilent C18 column); ESI-MS: calcd for $C_{34}H_{52}N_{10}O_{10}S_2$: 825.0 Da (average isotopes), (*m/z*) $[M+H]^+$: 825.3; found $[M+H]^+$: 825.2.

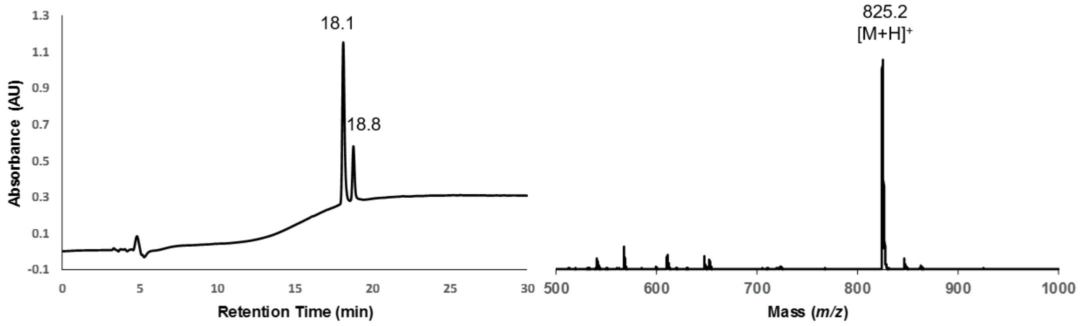

Figure S1. Left: UV trace from LC-MS analysis of peptide **7a**; Right: ESI-MS data of peptide **7a**.

Peptidyl Pen-Nbz 7b

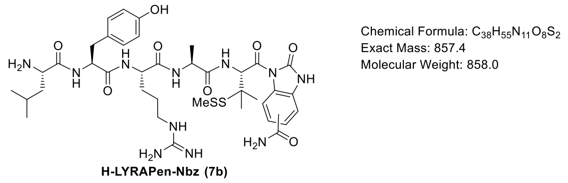

H-LYRAPen-Nbz (7b)

Peptide **7b** was prepared according to General Procedure **3.4** using Pioneer peptide synthesizer on a 0.02 mmol scale. Purification of the crude peptide using preparative HPLC (15 to 35% solvent B over 30min, Agilent Eclipse XDB-C18 column) afforded peptide **7b** as a white solid after lyophilization (9.5 mg, 60%). Analytical HPLC: t_R = 22.0 min (10 to 50% solvent B over 30min, Agilent C18 column); ESI-MS: calcd for

$C_{38}H_{55}N_{11}O_8S_2$: 825.0 Da (average isotopes), (*m/z*) $[M+H]^+$: 858.4; found $[M+H]^+$: 858.3.

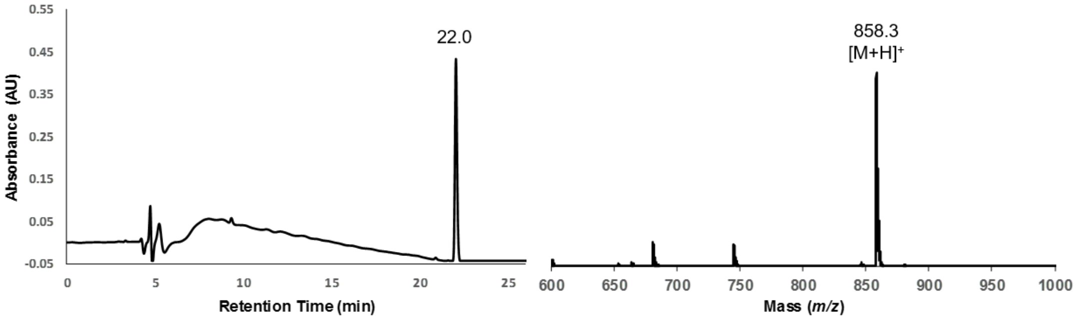

Figure S2. Left: UV trace from LC-MS analysis of peptide **7b**; Right: ESI-MS data of peptide **7b**.

<u>Peptidyl Pen-Nbz 7c</u>

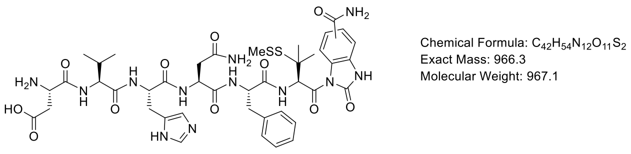

H-DVHNFPen-Nbz (7c)

Peptide **7c** (mixture of 3-/4-substitued Nbz isomers) was prepared according to General Procedure **3.4** using Pioneer peptide synthesizer on a 0.03 mmol scale. Purification of the crude peptide using preparative HPLC (15 to 40% solvent B over 30min, Agilent Eclipse XDB-C18 column) afforded peptide **7c** as a white solid after lyophilization (10.2 mg, 35%). Analytical HPLC: t_R = 20.6, 21.5 min (10 to 50% solvent B over 30min, Agilent C18 column); ESI-MS: calcd for $C_{42}H_{54}N_{12}O_{11}S_2$: 967.1 Da (average isotopes), (*m/z*) $[M+H]^+$: 967.4; found $[M+H]^+$: 967.4.

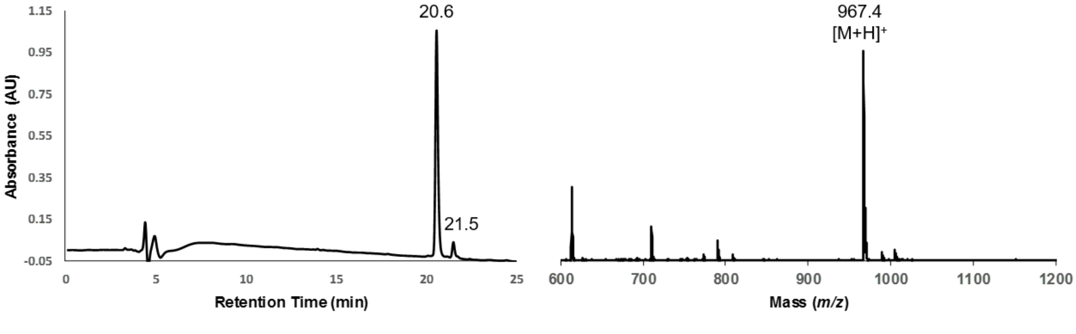

Figure S3. Left: UV trace from LC-MS analysis of peptide **7c**; Right: ESI-MS data of peptide **7c**.

Peptidyl Pen-Nbz 7d

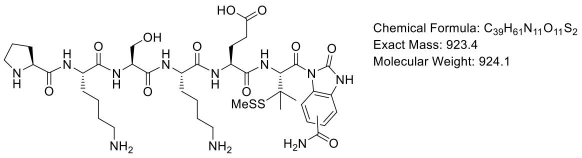

H-PKSKEPen-Nbz (7d)

Peptide **7d** (mixture of 3-/4-substitued Nbz isomers) was prepared according to General Procedure **3.4** using Pioneer peptide synthesizer on a 0.02 mmol scale. Purification of the crude peptide using preparative HPLC (10 to 30% solvent B over 30min, Agilent Eclipse XDB-C18 column) afforded peptide **7d** as a white solid after lyophilization (10.9 mg, 59%). Analytical HPLC: t_R = 16.3, 16.9 min (10 to 50% solvent B over 30min, Agilent C18 column); ESI-MS: calcd for $C_{39}H_{61}N_{11}O_{11}S_2$: 924.1 Da (average isotopes), (*m/z*) $[M+H]^+$: 924.4, $[M+2H]^{2+}$: 462.7; found $[M+H]^+$: 924.4, $[M+2H]^{2+}$: 462.9.

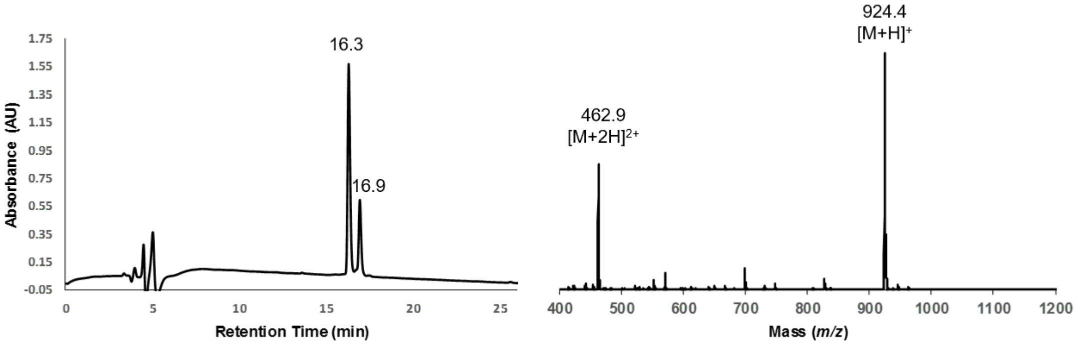

Figure S4. Left: UV trace from LC-MS analysis of peptide **7d**; Right: ESI-MS data of peptide **7d**.

peptidyl Val-Nbz 10

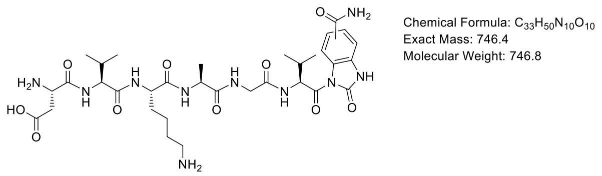

H-DVKAGV-Nbz (10)

Peptide **10** (mixture of 3-/4-substitued Nbz isomers) was prepared according to General Procedure **3.4** using Pioneer peptide synthesizer on a 0.02 mmol scale. Purification of the crude peptide using preparative HPLC (10 to 30% solvent B over 30min, Agilent Eclipse XDB-C18 column) afforded peptide **10** as a white solid after lyophilization (13.6 mg, 36%). Analytical HPLC: t_R = 17.0, 17.7 min (10 to 30% solvent B over 30min, Agilent C18 column); ESI-MS: calcd for $C_{33}H_{50}N_{10}O_{10}$: 746.8 Da (average isotopes), (*m/z*) $[M+H]^+$: 747.4; found $[M+H]^+$: 747.2.

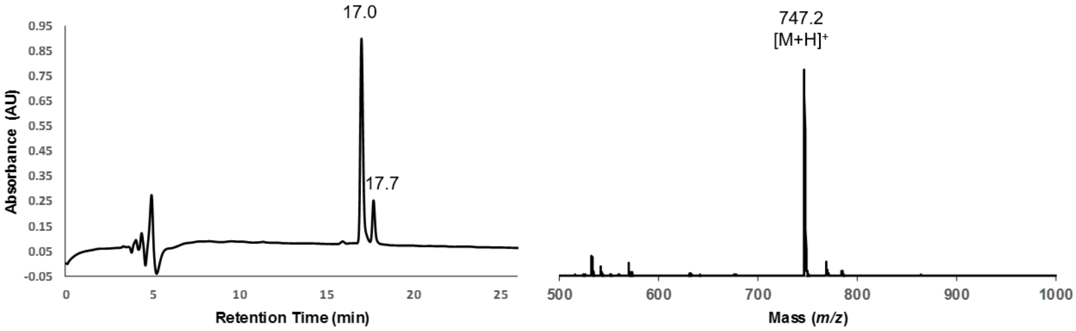

Figure S5. Left: UV trace from LC-MS analysis of peptide **10**; Right: ESI-MS data of peptide **10**.

Peptidyl Val-Nbz S6

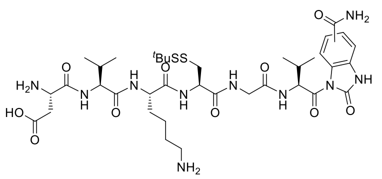

Chemical Formula: $C_{37}H_{58}N_{10}O_{10}S_2$
Exact Mass: 866.4
Molecular Weight: 867.1

H-DVKC(S^{t}Bu)GV-Nbz (S6)

Peptide **S6** was prepared according to General Procedure **3.4** using Pioneer peptide synthesizer on a 0.05 mmol scale. Purification of the crude peptide using preparative HPLC (10 to 30% solvent B over 30min, Agilent Eclipse XDB-C18 column) afforded peptide **S6** as a white solid after lyophilization (15.5 mg, 36%). Analytical HPLC: t_R = 21.8 min (10 to 30% solvent B over 30min, Agilent C18 column); ESI-MS: calcd for $C_{37}H_{58}N_{10}O_{10}S_2$: 867.1 Da (average isotopes), (*m/z*) $[M+H]^+$: 867.4; found $[M+H]^+$: 867.4.

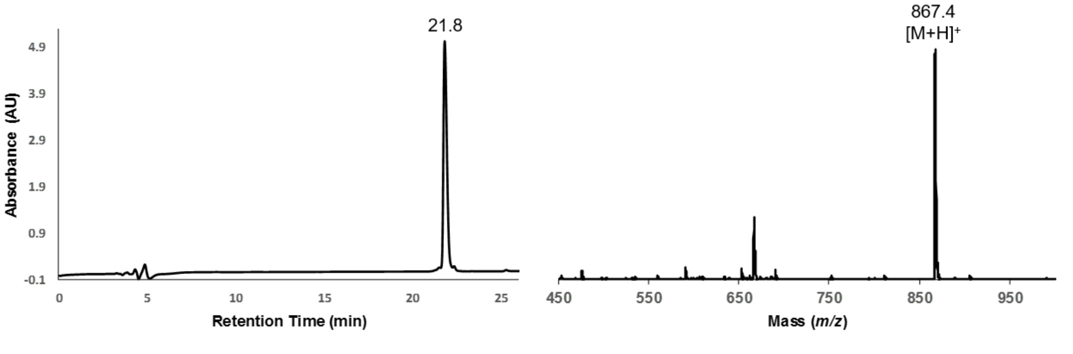

Figure S6. Left: UV trace from LC-MS analysis of peptide **S6**; Right: ESI-MS data of peptide **S6**.

Peptidyl Cys-Nbz S7

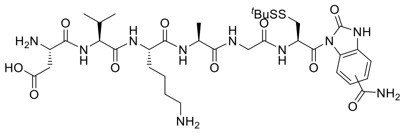

Chemical Formula: $C_{35}H_{54}N_{10}O_{10}S_2$
Exact Mass: 838.3
Molecular Weight: 839.0

H-DVKAGC(S^{t}Bu)-Nbz (S7)

Peptide **S7** (mixture of 3-/4-substitued Nbz isomers) was prepared according to General

Procedure **3.4** using Pioneer peptide synthesizer on a 0.02 mmol scale. Purification of the crude peptide using preparative HPLC (15 to 30% solvent B over 30min, Agilent Eclipse XDB-C18 column) afforded peptide **S7** as a white solid after lyophilization (4.2 mg, 24%). Analytical HPLC: t_R = 19.8, 20.3 min (10 to 50% solvent B over 30min, Agilent C18 column); ESI-MS: calcd for $C_{37}H_{50}N_8O_8S_2$: 839.0 Da (average isotopes), (*m/z*) $[M+H]^+$: 839.4; found $[M+H]^+$: 839.3.

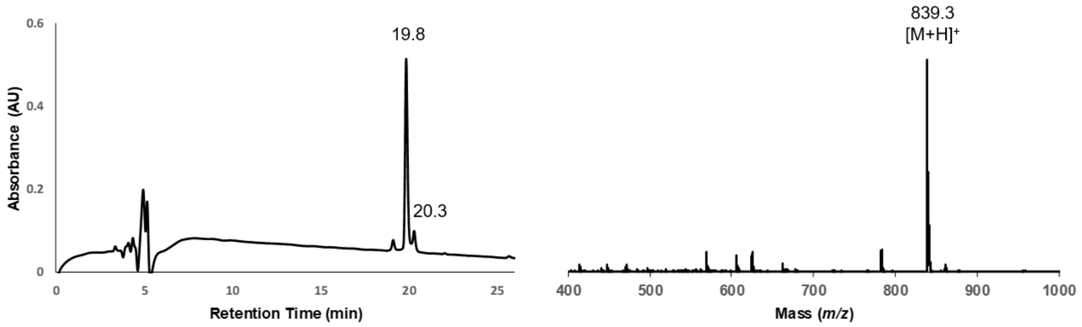

Figure S7. Left: UV trace from LC-MS analysis of peptide **S7**; Right: ESI-MS data of peptide **S7**.

<u>Peptide 8a</u>

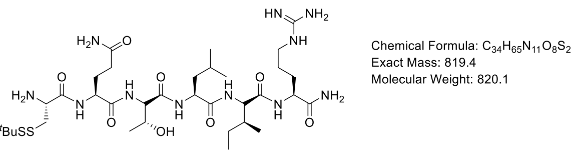

H-C(S^{t}Bu)QTLIR-NH$_2$ (8a)

Peptide **8a** was prepared according to General Procedure **3.3** using CS Bio peptide synthesizer on a 0.05 mmol scale. Purification of the crude peptide using preparative HPLC (10 to 30% solvent B over 30min, Agilent Eclipse XDB-C18 column) afforded peptide **8a** as a white solid after lyophilization (27.1 mg, 66%). Analytical HPLC: t_R = 19.6 min (10 to 40% solventB over 30min, Agilent C18 column); ESI-MS: calcd for $C_{34}H_{65}N_{11}O_8S_2$: 820.1 Da (average isotopes), (*m/z*) $[M+H]^+$: 820.5; found $[M+H]^+$: 820.6.

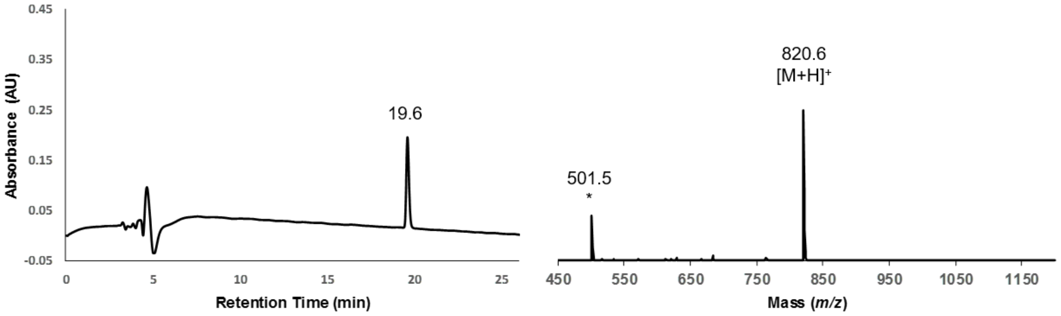

Figure S8. Left: UV trace from LC-MS analysis of peptide **8a**; Right: ESI-MS data of peptide **8a**. (* Denotes the peaks of fragmentation during ionization in mass spectrometer)

Peptide 8b

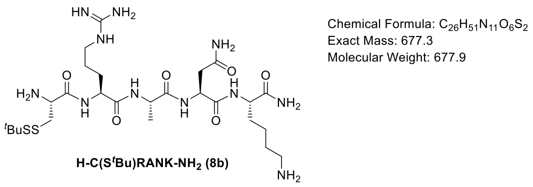

Peptide **8b** was prepared according to General Procedure **3.3** using Pioneer peptide synthesizer on a 0.02 mmol scale. Purification of the crude peptide using preparative HPLC (10 to 30% solvent B over 30min, Agilent Eclipse XDB-C18 column) afforded peptide **8b** as a white solid after lyophilization (13.6 mg, 36%). Analytical HPLC: t_R = 18.1 min (10 to 30% solvent B over 30min, Agilent C18 column); ESI-MS: calcd for $C_{26}H_{51}N_{11}O_6S_2$: 677.9 Da (average isotopes), (*m/z*) $[M+H]^+$: 678.4; found $[M+H]^+$: 678.2.

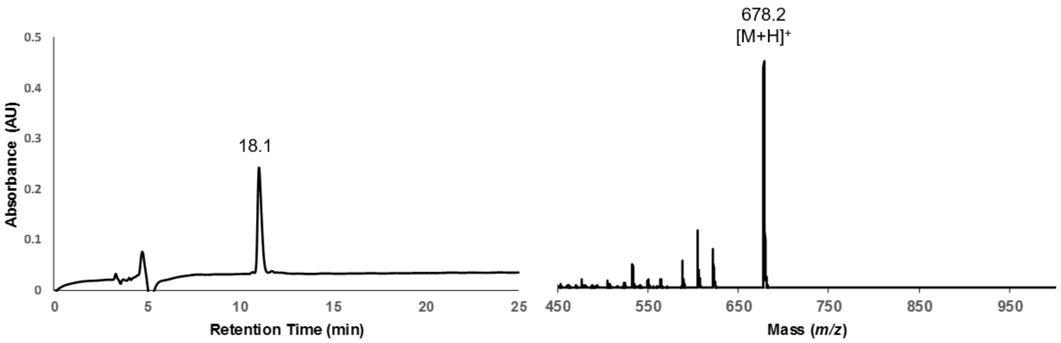

Figure S9. Left: UV trace from LC-MS analysis of peptide **8b**; Right: ESI-MS data of peptide **8b**.

Peptide 8c

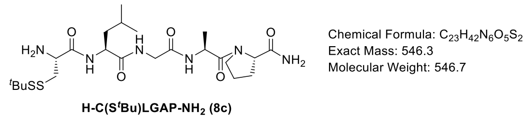

Peptide **8c** was prepared according to General Procedure **3.3** using Pioneer peptide

synthesizer on a 0.05 mmol scale. Purification of the crude peptide using preparative HPLC (10 to 30% solvent B over 30min, Agilent Eclipse XDB-C18 column) afforded peptide **8c** as a white solid after lyophilization (11.2 mg, 41%). Analytical HPLC: t_R = 13.8 min (20 to 50% solvent B over 30min, Agilent C18 column); ESI-MS: calcd for $C_{23}H_{42}N_6O_5S_2$: 546.7 Da (average isotopes), (*m/z*) $[M+H]^+$: 547.3; found $[M+H]^+$: 547.1.

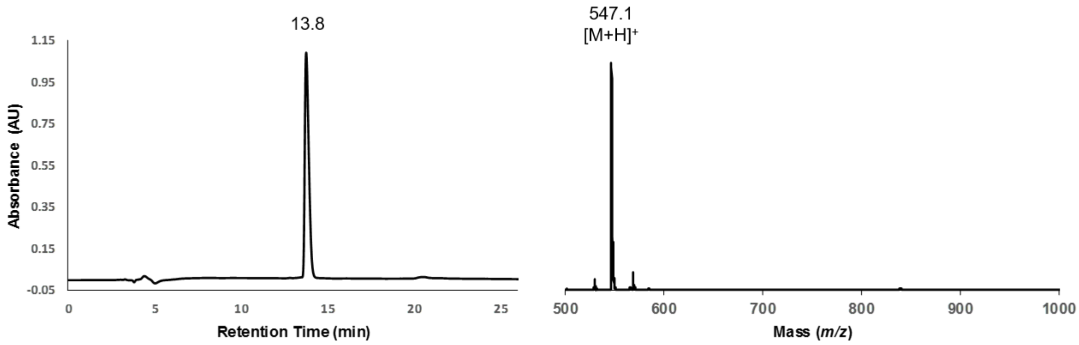

Figure S10. Left: UV trace from LC-MS analysis of peptide **8c**; Right: ESI-MS data of peptide **8c**.

Peptide 8d

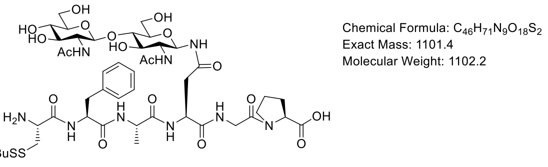

chitobiose
H-C(S^{t}Bu)FANGP-NH$_2$ (8d)

Peptide **8d** was prepared according to General Procedure **3.3** using Pioneer peptide synthesizer on a 0.05 mmol scale. Purification of the crude peptide using preparative HPLC (10 to 30% solvent B over 30min, Agilent Eclipse XDB-C18 column) afforded peptide **8d** as a white solid after lyophilization (6.1 mg, 11%). Analytical HPLC: t_R = 19.3 min (10 to 50% solvent B over 30min, Agilent C18 column); ESI-MS: calcd for $C_{46}H_{71}N_9O_{18}S_2$: 1102.2 Da (average isotopes), (*m/z*) $[M+H]^+$: 1102.4; found $[M+H]^+$: 1102.3.

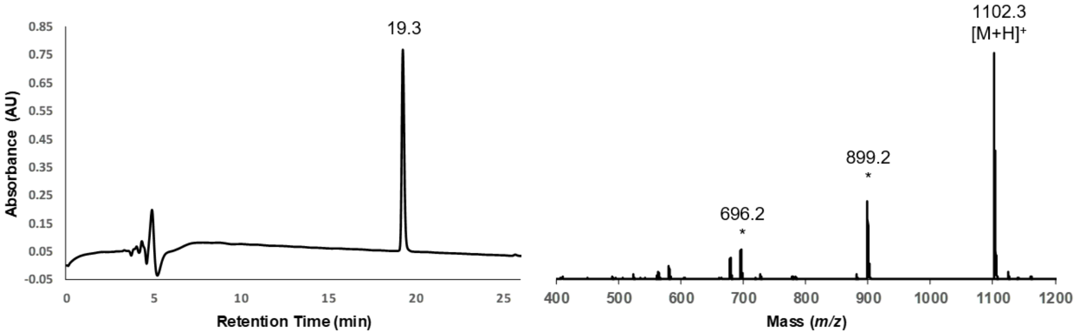

Figure S11. Left: UV trace from LC-MS analysis of peptide **8d**; Right: ESI-MS data of peptide **8d**. (* Denotes the peaks of fragmentation during ionization in mass spectrometer)

Peptide 8e

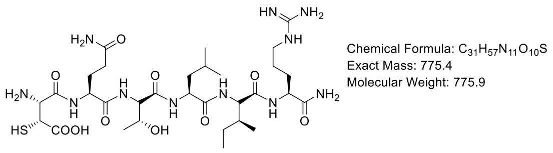

H-D(SH)QTLIR-NH$_2$ (8e)

Peptide **8e** was prepared according to General Procedure **3.3** using Pioneer peptide synthesizer on a 0.02 mmol scale. Purification of the crude peptide using preparative HPLC (10 to 30% solvent B over 30min, Agilent Eclipse XDB-C18 column) afforded peptide **8e** as a white solid after lyophilization (13.6 mg, 36%). Analytical HPLC: t_R = 17.4 min (10 to 30% solvent B over 30min, Agilent C18 column); ESI-MS: calcd for $C_{31}H_{57}N_{11}O_{10}S$: 775.9 Da (average isotopes), (*m/z*) $[M+H]^+$: 776.4; found $[M+H]^+$: 776.2.

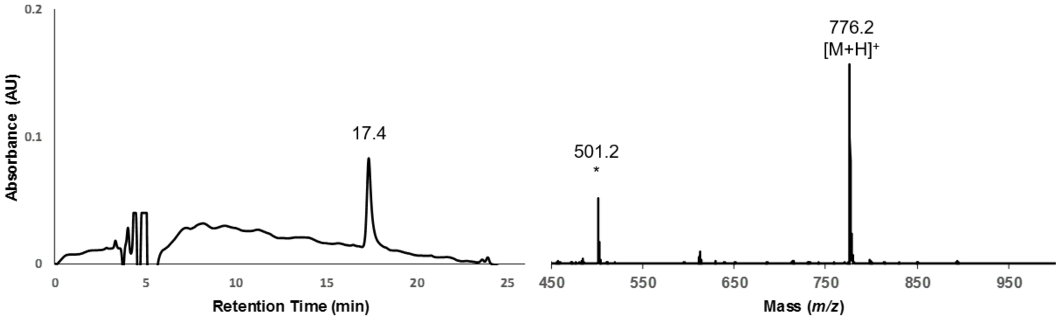

Figure S12. Left: UV trace from LC-MS analysis of peptide **8e**; Right: ESI-MS data of peptide **8e**. (* Denotes the peaks of fragmentation during ionization in mass spectrometer)

Peptide 8f

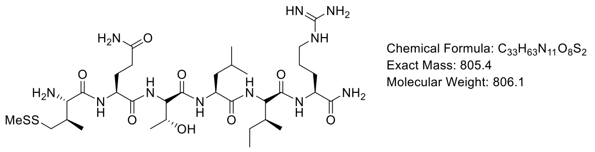

H-V($^{\beta}$SSMe)QTLIR-NH$_2$ (8f)

Peptide **8f** was prepared according to General Procedure **3.3** using Pioneer peptide synthesizer on a 0.02 mmol scale. Purification of the crude peptide using preparative HPLC (10 to 30% solvent B over 30min, Agilent Eclipse XDB-C18 column) afforded peptide **8f** as a white solid after lyophilization (4.5 mg, 28%). Analytical HPLC: t_R = 18.6 min (10 to 50% solvent B over 30min, Agilent C18 column); ESI-MS: calcd for $C_{33}H_{63}N_{11}O_8S$: 806.1 Da (average isotopes), (*m/z*) $[M+H]^+$: 806.4; found $[M+H]^+$: 806.3.

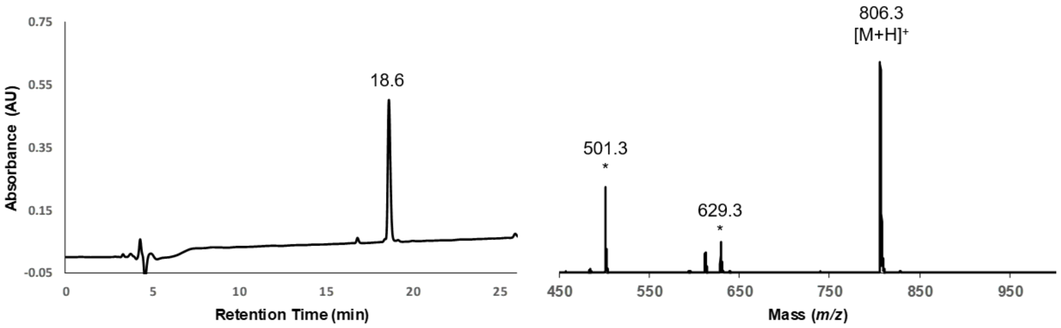

Figure S13. Left: UV trace from LC-MS analysis of peptide **8f**; Right: ESI-MS data of peptide **8f**. (* Denotes the peaks of fragmentation during ionization in mass spectrometer)

Peptide 8g

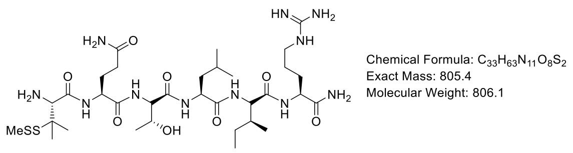

H-V($^{\beta}$SSMe)QTLIR-NH$_2$ (8g)

Peptide **8g** was prepared according to General Procedure **3.3** using Pioneer peptide synthesizer on a 0.05 mmol scale. Purification of the crude peptide using preparative HPLC (10 to 30% solvent B over 30min, Agilent Eclipse XDB-C18 column) afforded peptide **8g** as a white solid after lyophilization (10.5 mg, 26%). Analytical HPLC: t_R = 9.3 min (20 to 50% solvent B over 30min, Agilent C18 column); ESI-MS: calcd for $C_{33}H_{63}N_{11}O_8S$: 806.1 Da (average isotopes), (*m/z*) $[M+H]^+$: 806.4; found $[M+H]^+$: 806.3.

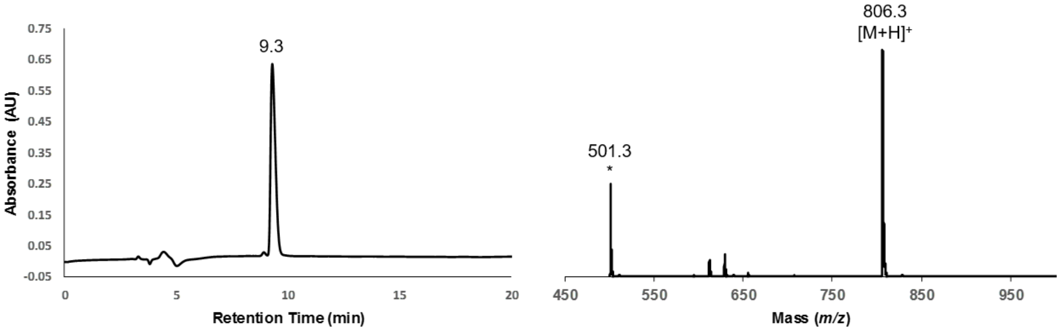

Figure S14. Left: UV trace from LC-MS analysis of peptide **8g**; Right: ESI-MS data of peptide **8g**. (* Denotes the peaks of fragmentation during ionization in mass spectrometer)

<u>Peptide S8</u>

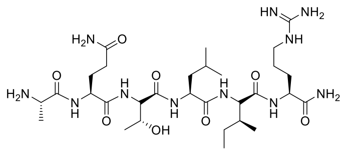

Chemical Formula: $C_{30}H_{57}N_{11}O_8$
Exact Mass: 699.4
Molecular Weight: 699.9

H-AQTLIR-NH$_2$ (S8)

Peptide **S8** was prepared according to General Procedure **3.3** using Pioneer peptide synthesizer on a 0.05 mmol scale. Purification of the crude peptide using preparative HPLC (10 to 30% solvent B over 30min, Agilent Eclipse XDB-C18 column) afforded peptide **S8** as a white solid after lyophilization (10.5 mg, 30%). Analytical HPLC: t_R = 15.4 min (5 to 50% B over solvent 30min, Agilent C18 column); ESI-MS: calcd for $C_{30}H_{57}N_{11}O_8$: 699.9 Da (average isotopes), (*m/z*) $[M+H]^+$: 700.5; found $[M+H]^+$: 700.2.

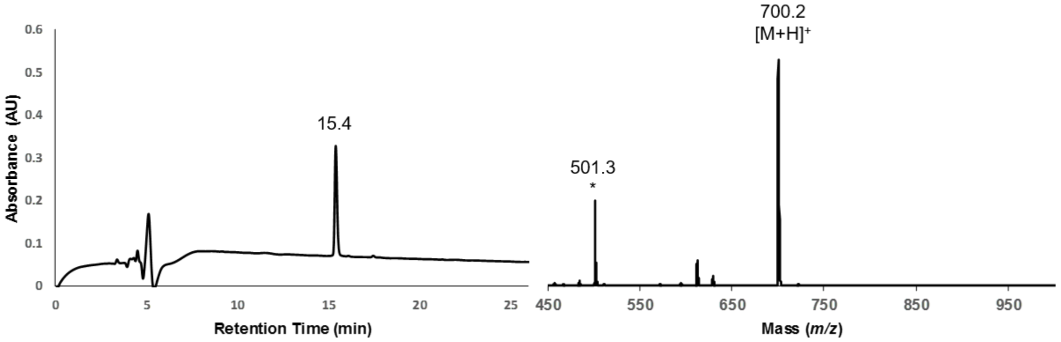

Figure S15. Left: UV trace from LC-MS analysis of peptide **S8**; Right: ESI-MS data of peptide **S8**. (* Denotes the peaks of fragmentation during ionization in mass spectrometer)

<u>Peptide S9</u>

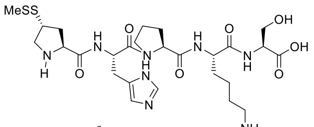

Chemical Formula: $C_{26}H_{42}N_8O_7S_2$
Exact Mass: 642.3
Molecular Weight: 642.8

H-P($^{\beta}$SSMe)HPKS-OH (S9)

Peptide **S9** was prepared following our previous work.[S5] Analytical HPLC: t_R = 13.7 min (5 to 40% B over solvent 30min, Agilent C18 column); ESI-MS: calcd for $C_{26}H_{42}N_8O_7S_2$: 642.8 Da (average isotopes), (m/z) [M+H]+: 643.3; found [M+H]+: 643.2.

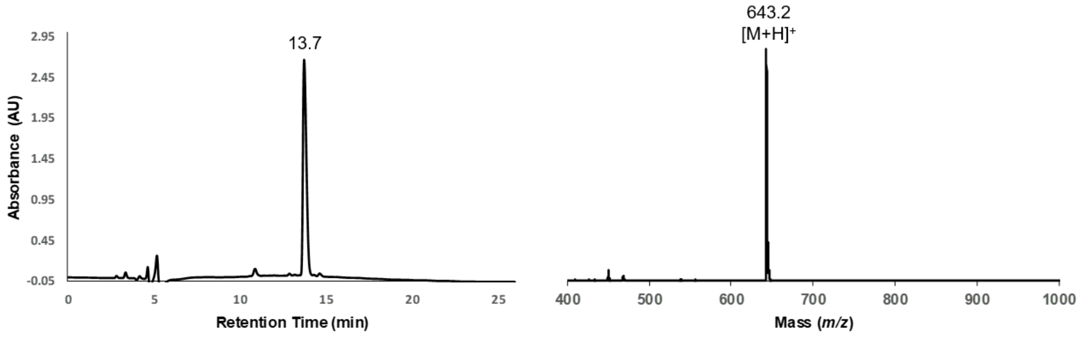

Figure S16. Left: UV trace from LC-MS analysis of peptide **S9**; Right: ESI-MS data of peptide **S9**.

<u>Peptidyl Pen-Nbz 7h</u>

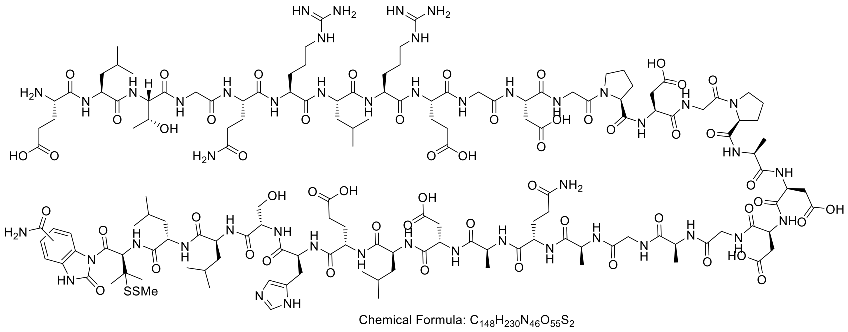

7h

Peptide **7h** was prepared according to General Procedure **3.4** using CS Bio peptide synthesizer on a 0.03 mmol scale. Purification of the crude peptide using preparative HPLC (25 to 35% solvent B over 30min, Proto-300 C4 column) afforded peptide **7h** as a white solid after lyophilization (18.4 mg, 17%). Analytical HPLC: t_R = 20.1 min (20 to 40% solvent B over 30min, Proto-300 C4 column); ESI-MS: calcd for $C_{148}H_{230}N_{46}O_{55}S_2$: 3597.9 Da (average isotopes), (*m/z*) $[M+2H]^{2+}$: 1799.3, $[M+3H]^{3+}$: 1199.9; found $[M+2H]^{2+}$: 1799.7, $[M+3H]^{3+}$:1200.1.

[S5] Y. Gui, L. Qiu, Y. Li, H. Li and S. Dong, *J. Am. Chem. Soc.*, 2016, **138**, 4890-4899.

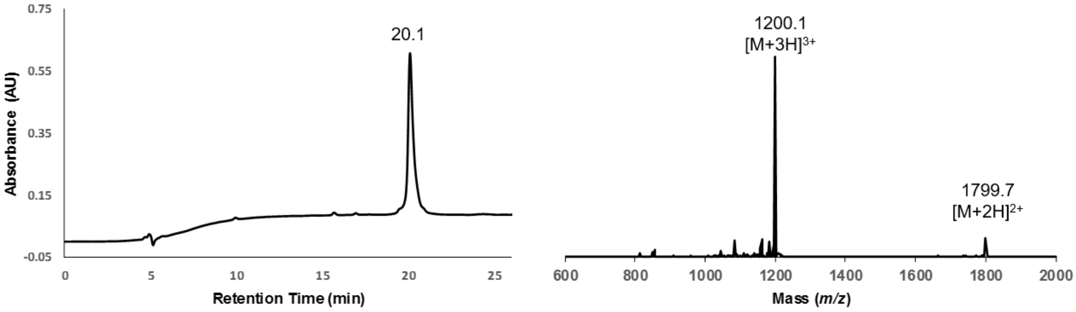

Figure S17. Left: UV trace from LC-MS analysis of peptide **7h**; Right: ESI-MS data of peptide **7h**.

Peptide 8h

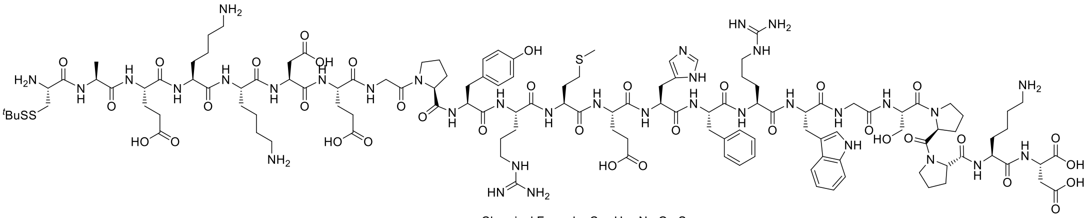

8h Chemical Formula: $C_{125}H_{187}N_{35}O_{36}S_3$
Exact Mass: 2850.3
Molecular Weight: 2852.3

Peptide **8h** was prepared according to General Procedure **3.3** using CS Bio peptide synthesizer on a 0.05 mmol scale. Purification of the crude peptide using preparative HPLC (15 to 30% solvent B over 30min, Beim Brueckle C4 column) afforded peptide **8h** as a white solid after lyophilization (64.4 mg, 65%). Analytical HPLC: t_R = 9.7 min (20 to 40% solvent B over 30min, Beim Brueckle C4 column); ESI-MS: calcd for $C_{125}H_{187}N_{35}O_{36}S_3$: 2852.3 Da (average isotopes), (*m/z*) $[M+2H]^{2+}$: 1426.7, $[M+3H]^{3+}$: 951.4, $[M+4H]^{4+}$: 713.8; found: $[M+2H]^{2+}$: 1426.7, $[M+3H]^{3+}$: 951.5, $[M+4H]^{4+}$: 713.9.

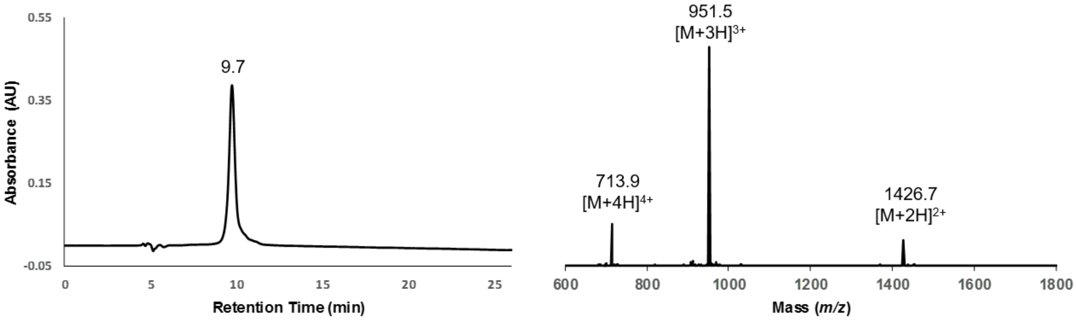

Figure S18. Left: UV trace from LC-MS analysis of peptide **8h**; Right: ESI-MS data of peptide **8h**.

Peptidyl thioester 13

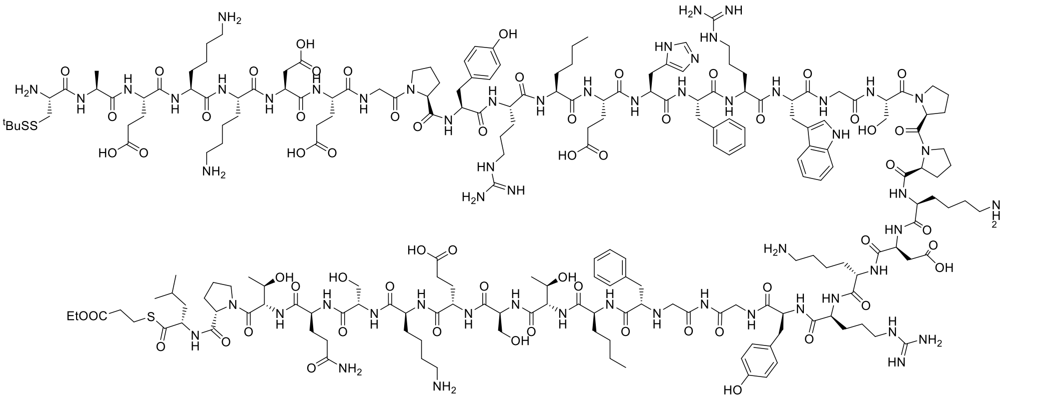

13

Side-chain protected peptide Cys[34]-Pro[71] with Boc-protection at the *N*-terminus was synthesized using CS Bio peptide synthesizer on a 0.05 mmol scale. The resin was cleaved using DCM/TFE/AcOH (3:1:1, *v*/*v*/*v*) for three times (7 mL, 7 mL and 6 mL). The filtrate was concentrated under an argon atmosphere, and lyophilized to remove the residual acid. To the obtained powder (1.0 equiv) was added leucine thioester (2.0 equiv) and HATU (2.0 equiv), and the mixture was dissolved in anhydrous DCM, followed by the addition of DIEA (4.0 equiv) at 0 °C. The reaction mixture was stirred at room temperature for 30 min. After removal of the solvent under a nitrogen atmosphere, the resulting residue was treated with a solution of TFA/H_2O/TIPS (95:2.5:2.5, *v*/*v*/*v*) for 2 hours, and then concentrated under a nitrogen atmosphere. The residue was washed with cold diethyl ether to afford a white solid, which was then dissolved in a mixture of acetonitrile and water containing 5% of acetic acid. The resulting solution was ready for HPLC purification after filtration. Purification of the crude peptide using preparative HPLC (15 to 30% solvent B over 30min, Proto-300 C4 column) afforded peptide **13** as a white solid after lyophilization (23.6 mg, 10%). Analytical HPLC: t_R = 19.6 min (20 to 40% solvent B over 30min, Proto-300 C4 column); ESI-MS: calcd for $C_{212}H_{325}N_{57}O_{61}S_2$: 4744.5 Da (average isotopes), (*m/z*) $[M+3H]^{3+}$: 1582.1, $[M+4H]^{4+}$: 1186.8, $[M+5H]^{5+}$: 949.7; found: $[M+3H]^{3+}$: 1582.3, $[M+4H]^{4+}$: 1186.9, $[M+5H]^{5+}$: 949.9.

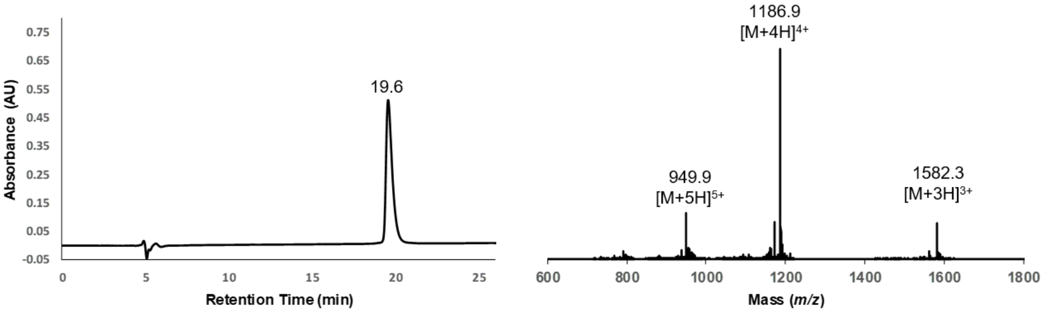

Figure S19. Left: UV trace from LC-MS analysis of peptide **13**; Right: ESI-MS data of peptide **13**.

Peptide 15b

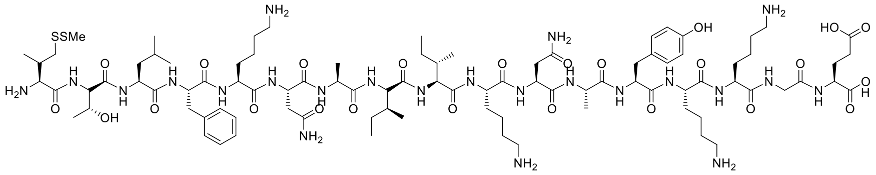

15b Chemical Formula: $C_{91}H_{151}N_{23}O_{24}S_2$
Exact Mass: 2014.1
Molecular Weight: 2015.5

Peptide **15b** was prepared according to General Procedure **3.3** using CS Bio peptide synthesizer on a 0.05 mmol scale, and the *N*-terminal thio-valine derivative was coupled manually using Boc-Val($^{\gamma}$SSMe)-OH[S2] (1.5 equiv) and HATU (1.5 equiv) for 30 min (×2). Purification of the crude peptide using preparative HPLC (15 to 30% solvent B over 30min, Beim Brueckle C4 column) afforded peptide **15b** as a white solid after lyophilization (35.4mg, 59%). Analytical HPLC: t_R = 18.5 min (15 to 45% solvent B over 30min, Beim Brueckle C4 column); ESI-MS: calcd for $C_{91}H_{151}N_{23}O_{24}S_2$: 2015.5 Da (average isotopes), (*m/z*) $[M+2H]^{2+}$: 1008.0, $[M+3H]^{3+}$: 672.3; found: $[M+2H]^{2+}$: 1008.1, $[M+3H]^{3+}$: 672.6.

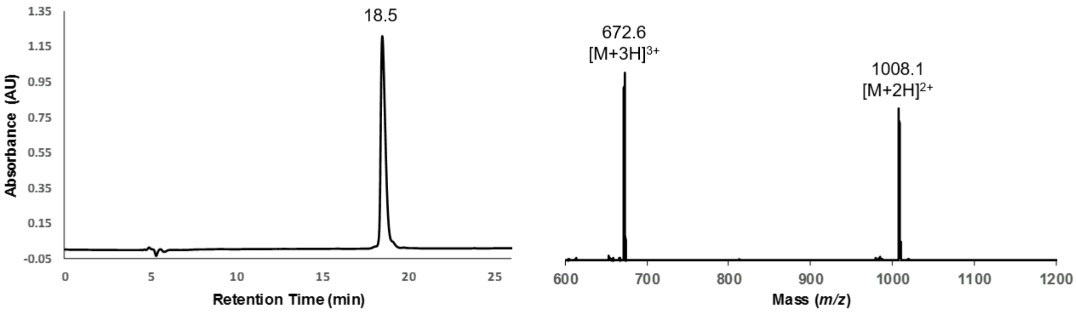

Figure S20. Left: UV trace from LC-MS analysis of peptide **15b**; Right: ESI-MS data of peptide **15b**.

Peptidyl Pen-Nbz 18

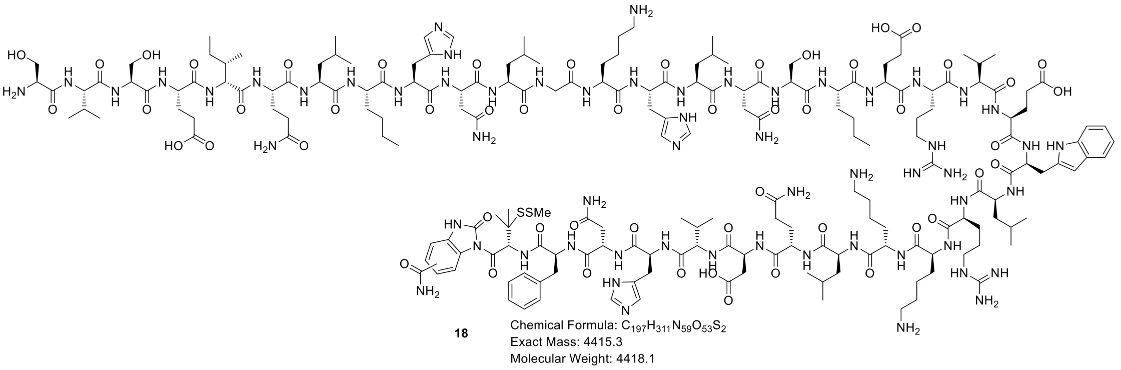

Peptide **18** was prepared according to General Procedure **3.4** using CS Bio peptide synthesizer on a 0.05 mmol scale. Asn^{16}Ser^{17}was installed manually using Fmoc-Asn(Trt)-Ser($\psi^{Me,Me}$Pro)-OH as the building block. After peptide elongation, the resin was treated with a *p*-nitrophenylchloroformate solution (50 mM in dry DCM) for 2 hours at room temperature. Purification of the crude peptide using preparative HPLC (30 to 40% solvent B over 30min, Proto-300 C4 column) afforded peptide **18** as a white solid after lyophilization (21.5 mg, 13%). Analytical HPLC: t_R = 28.6 min (20 to 40% solvent B over 30min, Beim Brueckle C4 column); ESI-MS: calcd for $C_{197}H_{311}N_{59}O_{53}S_2$: 4418.1 Da (average isotopes), (*m/z*) $[M+3H]^{3+}$: 1473.4, $[M+4H]^{4+}$: 1105.3, $[M+5H]^{5+}$: 884.5; found: $[M+3H]^{3+}$: 1473.3, $[M+4H]^{4+}$: 1105.2, $[M+5H]^{5+}$: 884.4.

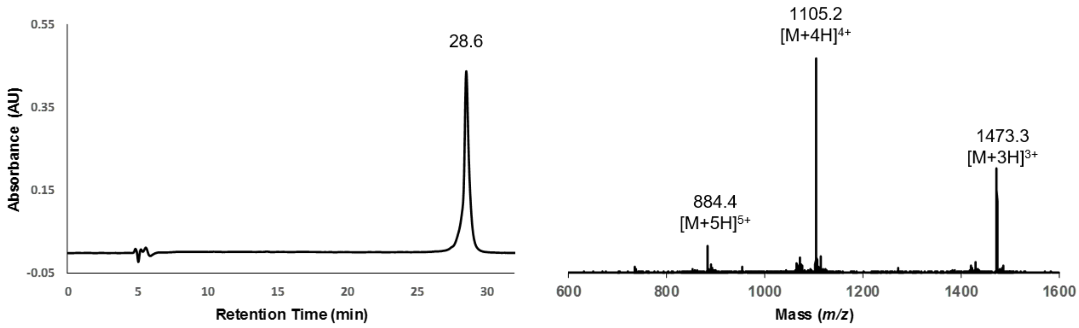

Figure S21. Left: UV trace from LC-MS analysis of peptide **18**; Right: ESI-MS data of peptide **18**.

Peptidyl thioester 19

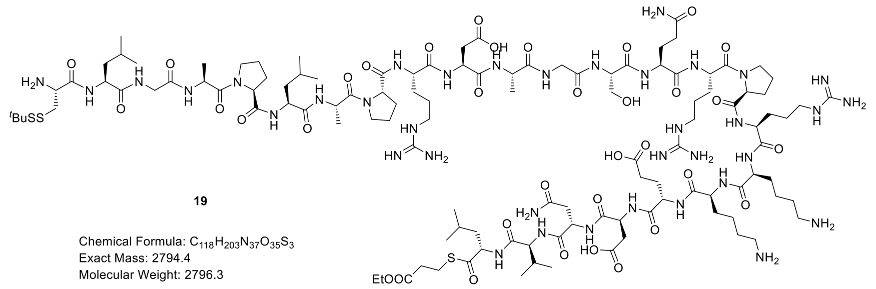

19

Side-chain protected peptide Cys[36]-Leu[57] with Boc-protection at the *N*-terminus was synthesized using CS Bio peptide synthesizer on a 0.1 mmol scale. The resin was cleaved using DCM/TFE/AcOH (3:1:1, *v/v/v*) for three times (7 mL, 7 mL and 6 mL). The filtrate was concentrated under a nitrogen atmosphere, and lyophilized to remove the residual acid. To a solution of the obtained powder (1.0 equiv) and 6-Chloro-benzotriazole-1-yloxy-tris-pyrrolidinophosphonium hexafluorophosphate (PyClock, 3.0 equiv) in anhydrous DMF, ethyl 3-mercaptopropionate (30 equiv) was added followed by the addition of DIEA (5.0 equiv) at -20 °C. The reaction mixture was stirred at the same temperature for 2 hours.[S6] After removal of the solvent under lyophilization, the resulting residue was treated with a solution of TFA/H_2O/TIPS (95:2.5:2.5, *v/v/v*) for 2 hours, and then concentrated under a nitrogen atmosphere. The residue was washed with cold diethyl ether to afford a white solid, which was then dissolved in a mixture of acetonitrile and water containing 5% of acetic acid. The resulting solution was ready for HPLC purification after filtration. Purification of the crude peptide using preparative HPLC (20 to 35% solvent B over 30min, Proto-300 C4 column) afforded peptide **19** as a white solid after lyophilization (61.5 mg, 22%). Analytical HPLC: t_R = 20.9 min (20 to 40% solvent B over 30min, Proto-300 C4 column); ESI-MS: calcd for $C_{118}H_{203}N_{37}O_{35}S_3$: 2796.3 Da (average isotopes), (*m/z*) $[M+2H]^{2+}$: 1398.7, $[M+3H]^{3+}$: 932.8, $[M+4H]^{4+}$: 699.9; found: $[M+2H]^{2+}$: 1398.9, $[M+3H]^{3+}$: 932.7, $[M+4H]^{4+}$: 699.9.

[S6] Y. Kajihara, A. Yoshihara, K. Hirano, N. Yamamoto, *Carbohydr. Res.* **2006**, *341*, 1333-1340.

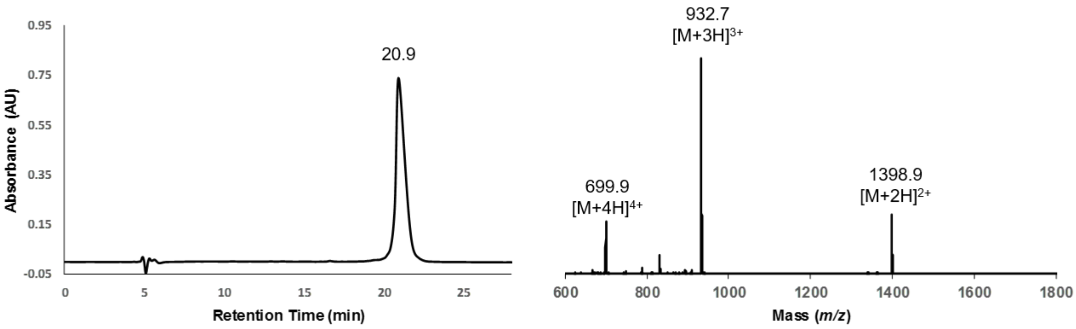

Figure S22. Left: UV trace from LC-MS analysis of peptide **19**; Right: ESI-MS data of peptide **19**.

Peptide 21

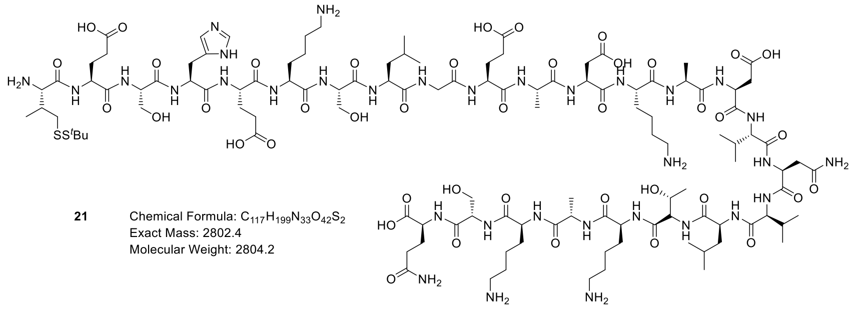

Peptide **21** was prepared according to General Procedure **3.3** using CS Bio peptide synthesizer on a 0.05 mmol scale, and the *N*-terminal thio-valine derivative was coupled manually using **S5** (1.5 equiv) for 30 min (×2). Purification of the crude peptide using preparative HPLC (15 to 30% solvent B over 30min, Proto-300 C4 column) afforded peptide **21** as a white solid after lyophilization (16.4mg, 13%). Analytical HPLC: t_R = 16.9 min (15 to 45% solvent B over 30min, Proto-300 C4 column); ESI-MS: calcd for $C_{117}H_{199}N_{33}O_{42}S_2$: 2804.2 Da (average isotopes), (*m/z*) $[M+2H]^{2+}$: 1402.7, $[M+3H]^{3+}$: 935.5, $[M+4H]^{4+}$: 701.9; found: $[M+2H]^{2+}$: 1402.8, $[M+3H]^{3+}$: 935.5, $[M+4H]^{4+}$: 701.9.

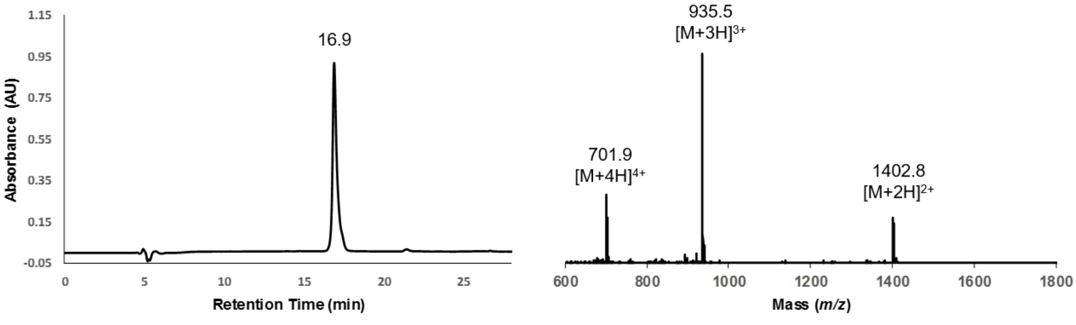

Figure S23. Left: UV trace from LC-MS analysis of peptide **21**; Right: ESI-MS data of peptide **21**.

V. Ligation Reactions Probing the Mechanistic Insight

Ligation reactions between S6 and 8a

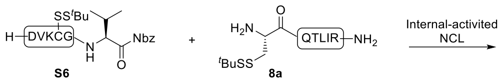

Peptidyl Val-Nbz **S6** (1.0 equiv) and cysteinyl peptide **8a** (1.2 equiv) were subjected to the ligation conditions following General Procedure **3.5** as described previously. The reaction was stirred at room temperature for 8 hours, and the progress was monitored with LC-MS. As shown in Figure S24, a complex mixture was generated with low conversion to the ligation product after 8 h.

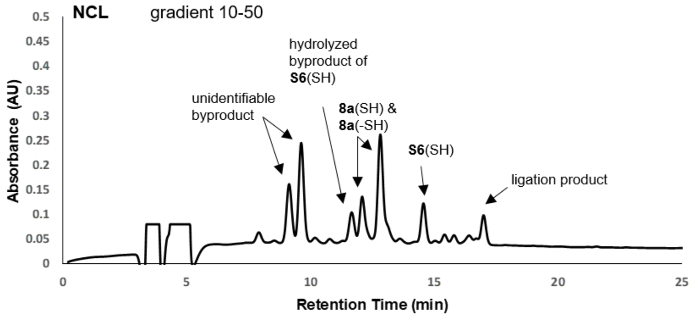

Figure S24. UV trace of the ligation reaction between peptides **S6** and **8a**.

Ligation reactions between S7 and 8a

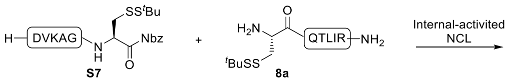

Peptidyl Cys-Nbz **S7** (1.0 equiv) and cysteinyl peptide **8a** (1.2 equiv) were subjected to the ligation conditions following General Procedure **3.5** as described previously. The reaction was stirred at room temperature for 2 hours, and the progress was monitored with LC-MS. As shown in Figure S25, large amount of **S7** decomposed after 2 hours, and only trace amount of ligation product was generated.

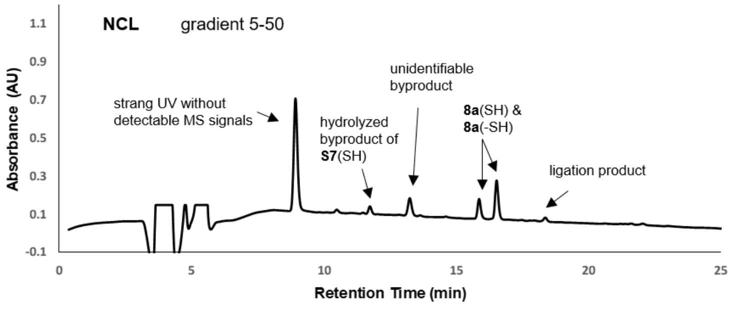

Figure S25. UV trace of the ligation reaction between peptides **S7** and **8a**.

Ligation reactions between 7a and S8

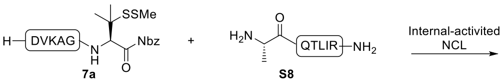

Peptidyl Pen-Nbz **7a** (1.0 equiv) and alanyl peptide **S8** (1.2 equiv) were subjected to the ligation conditions following General Procedure **3.5** as described previously. The reaction was stirred at room temperature for 8 hours, and the progress was monitored with LC-MS. As shown in Figure S26, large amount of byproduct was generated and trace amount of ligation product was observed.

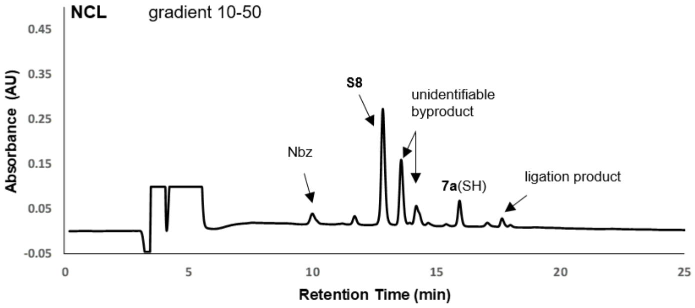

Figure S26. UV trace of the ligation reaction between peptides **7a** and **S8**.

7a in ligation buffer

Peptidyl Pen-Nbz **7a** (0.4 mg) was dissolved in 160 μL of ligation buffer, and monitored with LC-MS (5 to 30% B over solvent 30min, Agilent C18 column). As shown in Figure S27, the intermediate **7a'** gradually decomposes and hydrolizes over time if not intercepted with other reactants.

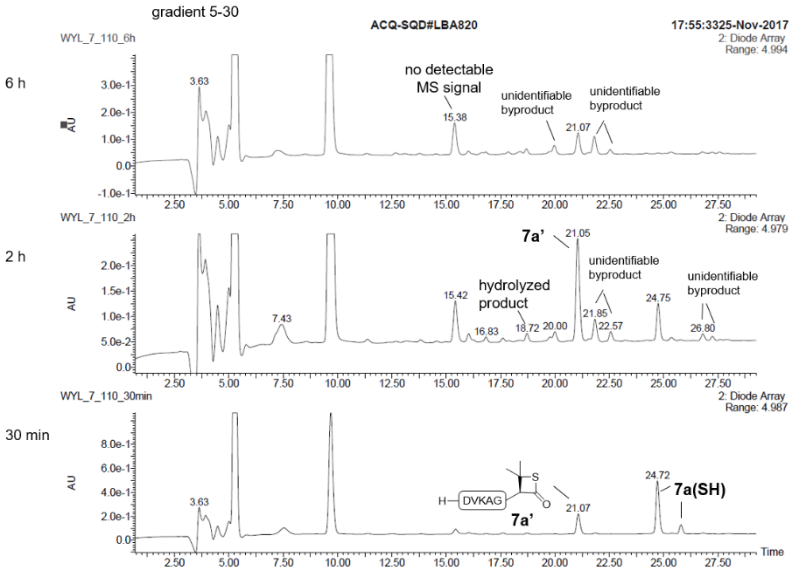

Figure S27. UV trace of peptide **7a** dissolved in ligation buffer.

VI. One-Pot Ligation and Desulfurization Reactions

One-pot ligation and desulfurization reactions between 7a and 8a

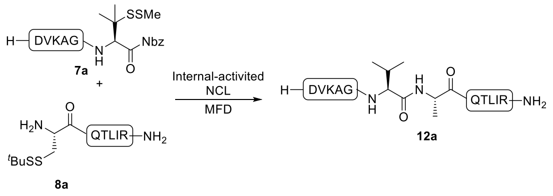

0.70 mg of peptidyl Nbz **7a** and 0.81 mg of cysteinyl peptide **8a** were subjected to the ligation conditions following General Procedure **3.5** as described previously. The reaction was stirred for 2 h at room temperature, followed by the addition of Bond-breaker® TCEP solution, ^{t}BuSH and VA-044 following General Procedure **3.7**. The reaction was stirred for another 3 h at 37 °C under an argon atmosphere, and quenched with 1.0 mL of $CH_3CN/H_2O/AcOH$ (5/90/5) solution. The resulting mixture was subjected to LC-MS analysis, and purified using preparative HPLC (10-30% solvent B over 30 min, Agilent Eclipse XDB-C18 column). The fractions containing pure peptide was concentrated affording **12a** (0.72 mg, 65%) as a white solid. Analytical HPLC for **12a**: t_R = 19.0 min (5 to 50% solvent B over 30min, Agilent C18 column); ESI-MS: calcd for $C_{55}H_{100}N_{18}O_{16}$: 1269.5 Da (average isotopes), (*m/z*) $[M+H]^+$: 1269.8, $[M+2H]^{2+}$: 635.4; found: $[M+H]^+$: 1269.5, $[M+2H]^{2+}$: 635.4.

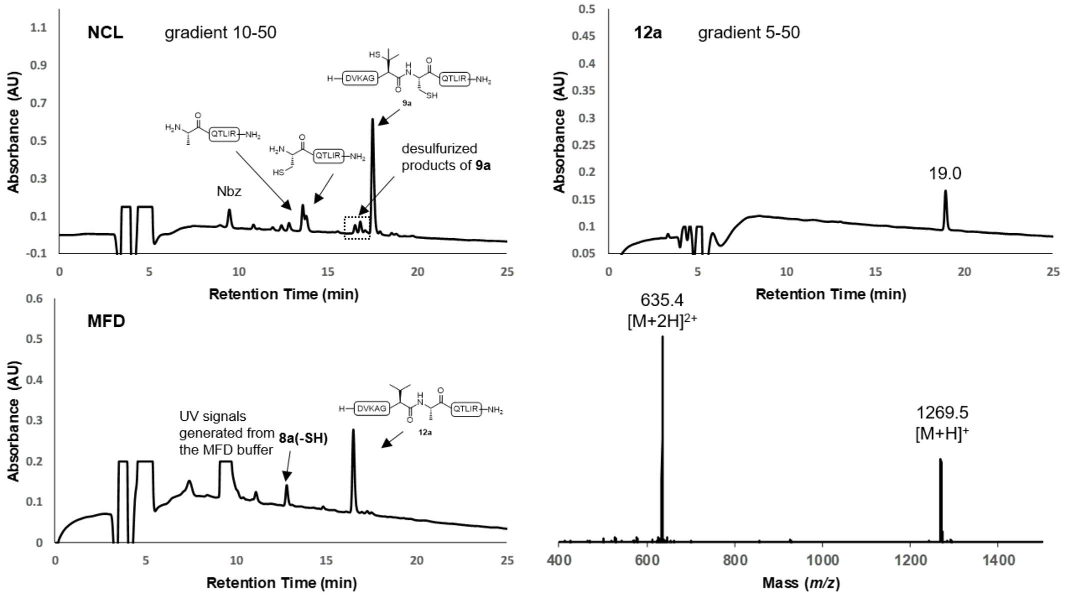

Figure S28. Top left: UV trace of ligation reaction between peptides **7a** and **8a**; Bottom left: UV trace of one-pot desulfurization following ligation; Top right: UV trace of purified **12a**; Bottom right: ESI-MS data of purified **12a**.

One-pot ligation and desulfurization reactions between 7b and 8b

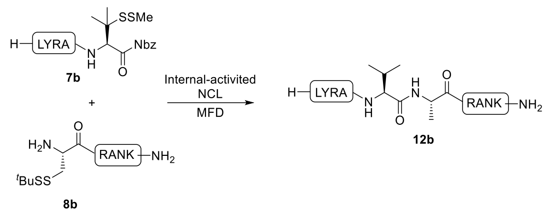

0.70 mg of peptidyl Nbz **7b** and 0.60 mg of cysteinyl peptide **8b** were subjected to the ligation conditions following General Procedure **3.5** as described previously. The reaction was stirred for 2 h at room temperature, followed by the addition of Bond-breaker® TCEP solution, ^{t}BuSH and VA-044 following General Procedure **3.7**. The reaction was stirred for another 3 h at 37 °C under an argon atmosphere, and quenched with 1.0 mL of $CH_3CN/H_2O/AcOH$ (5/90/5) solution. The resulting mixture was subjected to LC-MS analysis, and purified using preparative HPLC (10-30% solvent B over 30 min, Agilent Eclipse XDB-C18 column). The fractions containing pure peptide was concentrated affording **12b** (0.65 mg, 62%) as a white solid. Analytical HPLC for **12b**: t_R = 17.0 min (10 to 50% solvent B over 30min, Agilent C18 column); ESI-MS: calcd for $C_{51}H_{89}N_{19}O_{12}$: 1160.4 Da (average isotopes), (*m/z*) $[M+H]^+$: 1160.7, $[M+2H]^{2+}$: 580.9; found: $[M+H]^+$: 1160.4, $[M+2H]^{2+}$: 581.1.

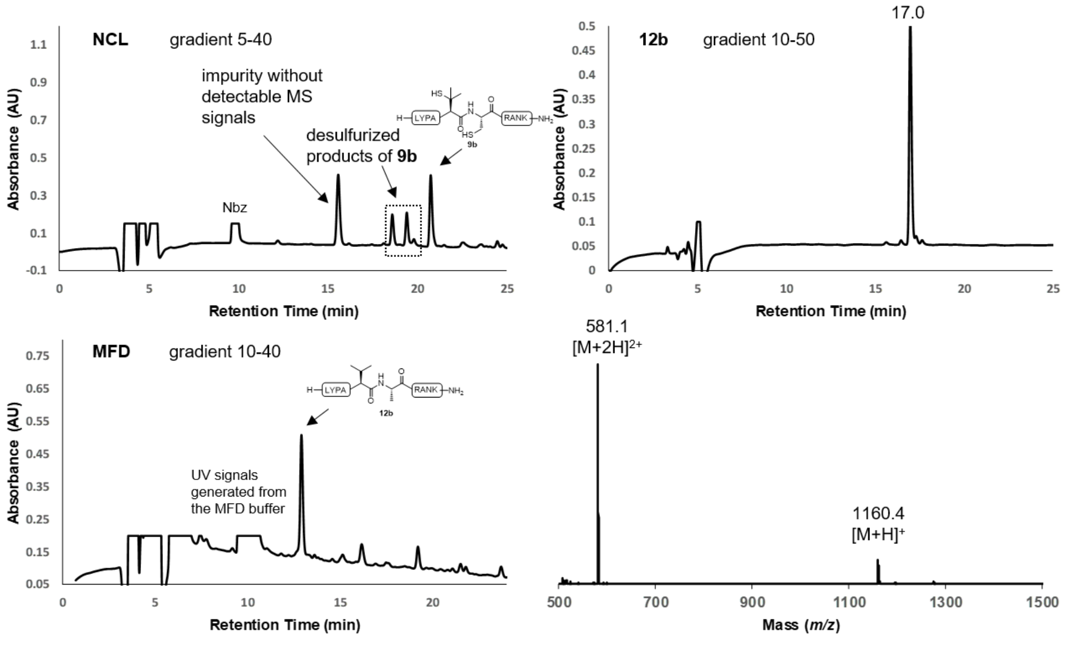

Figure S29. Top left: UV trace of ligation reaction between peptides **7b** and **8b**; Bottom left: UV trace of one-pot desulfurization following ligation; Top right: UV trace of purified **12b**; Bottom right: ESI-MS data of purified **12b**.

One-pot ligation and desulfurization reactions between 7c and 8c

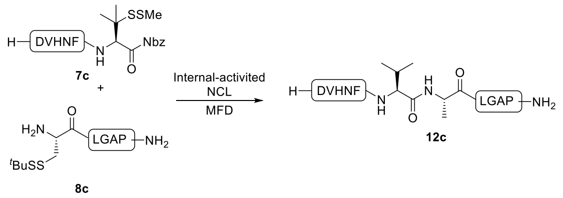

0.95 mg of peptidyl Nbz **7c** and 0.73 mg of cysteinyl peptide **8c** were subjected to the ligation conditions following General Procedure **3.5** as described previously. The reaction was stirred for 2 h at room temperature, followed by the addition of Bond-breaker® TCEP solution, ^{t}BuSH and VA-044 following General Procedure **3.7**. The reaction was stirred for another 3 h at 37 °C under an argon atmosphere, and quenched with 1.0 mL of CH_3CN/H_2O/AcOH (5/90/5) solution. The resulting mixture was subjected to LC-MS analysis, and purified using preparative HPLC (10-30% solvent B over 30 min, Agilent Eclipse XDB-C18 column). The fractions containing pure peptide was concentrated affording **12c** (0.63 mg, 50%) as a white solid. Analytical HPLC for **12c**: t_R = 18.4 min (10 to 50% solvent B over 30min, Agilent C18 column); ESI-MS: calcd for $C_{52}H_{79}N_{15}O_{14}$: 1138.3 Da (average isotopes), (*m/z*) $[M+H]^+$: 1138.6; found: $[M+H]^+$: 1138.6.

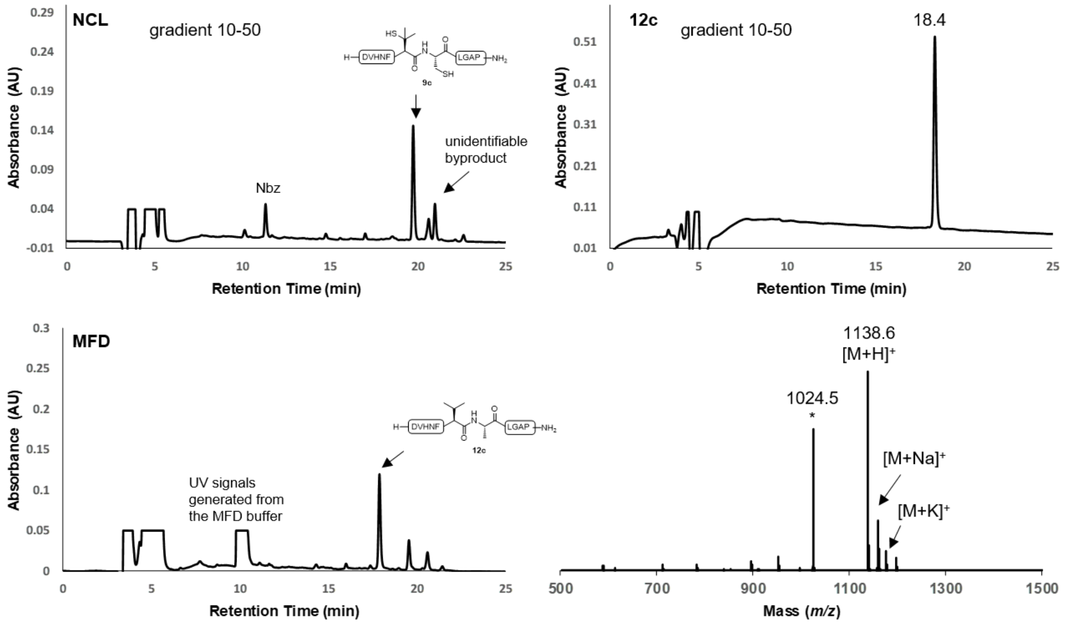

Figure S30. Top left: UV trace of ligation reaction between peptides **7c** and **8c**; Bottom left: UV trace of one-pot desulfurization following ligation; Top right: UV trace of purified **12c**; Bottom right: ESI-MS data of purified **12c**. (* Denotes the peaks of fragmentation during ionization in mass spectrometer)

One-pot ligation and desulfurization reactions between 7d and 8d

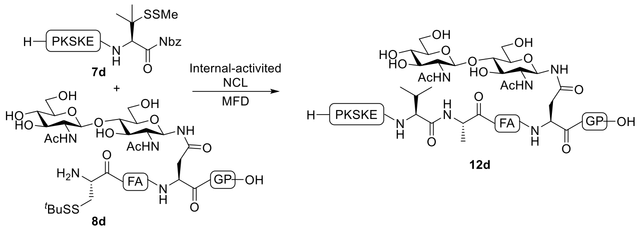

0.42 mg of peptidyl Nbz **7d** and 1.37 mg of cysteinyl peptide **8d** were subjected to the ligation conditions following General Procedure **3.5** as described previously. The reaction was stirred for 2 h at room temperature, followed by the addition of Bond-breaker® TCEP solution, ^{t}BuSH and VA-044 following General Procedure **3.7**. The reaction was stirred for another 3 h at 37 °C under an argon atmosphere, and quenched with 1.0 mL of $CH_3CN/H_2O/AcOH$ (5/90/5) solution. The resulting mixture was subjected to LC-MS analysis, and purified using preparative HPLC (10-30% solvent B over 30 min, Agilent Eclipse XDB-C18 column). The fractions containing pure peptide was concentrated affording **12d** (0.45 mg, 60%) as a white solid. Analytical HPLC for **12d**: t_R = 13.6 min (10 to 50% solvent B over 30min, Agilent C18 column); ESI-MS: calcd for $C_{72}H_{115}N_{17}O_{27}$: 1650.8 Da (average isotopes), (*m/z*) $[M+H]^+$: 1650.8, $[M+2H]^{2+}$: 825.9; found: $[M+H]^+$: 1651.8, $[M+2H]^{2+}$: 826.3.

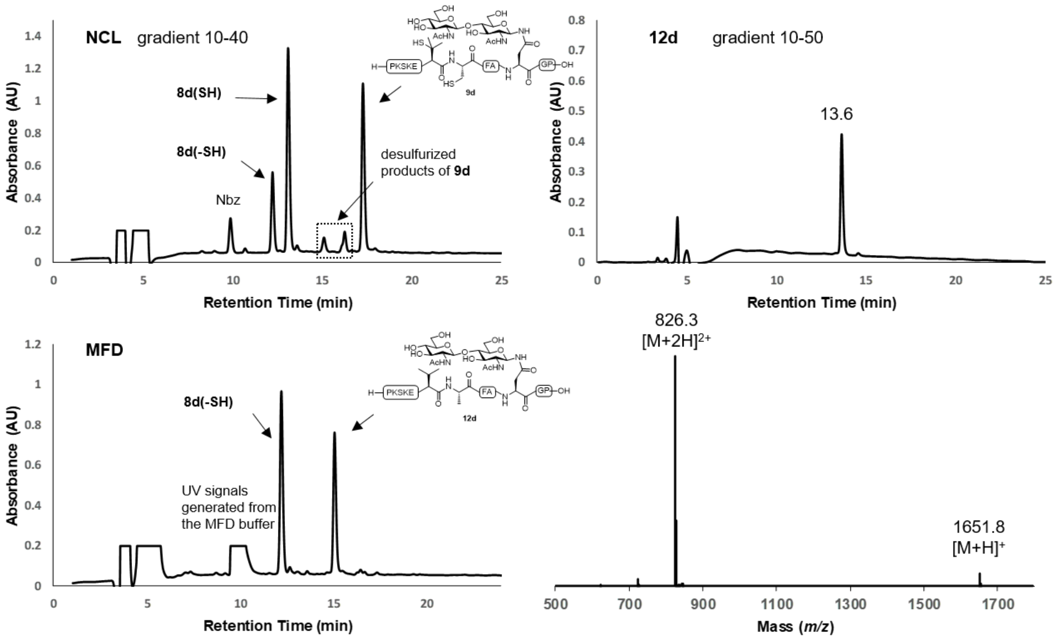

Figure S31. Top left: UV trace of ligation reaction between peptides **7d** and **8d**; Bottom left: UV trace of one-pot desulfurization following ligation; Top right: UV trace of purified **12d**; Bottom right: ESI-MS data of purified **12d**.

One-pot ligation and desulfurization reactions between 7a and 8e

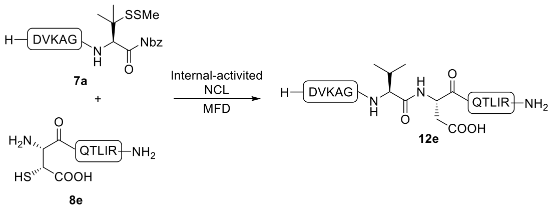

0.60 mg of peptidyl Nbz **7a** and 0.64 mg of cysteinyl peptide **8e** were subjected to the ligation conditions following General Procedure **3.5** as described previously. The reaction was stirred for 2 h at room temperature, followed by the addition of Bond-breaker® TCEP solution, *t*BuSH and ACVA following General Procedure **3.7**. The reaction was stirred for another 5 h at 37 °C under an argon atmosphere, and quenched with 1.0 mL of CH_3CN/H_2O/AcOH (5/90/5) solution. The resulting mixture was subjected to LC-MS analysis, and purified using preparative HPLC (10-30% solvent B over 30 min, Agilent Eclipse XDB-C18 column). The fractions containing pure peptide was concentrated affording **12e** (0.47 mg, 50%) as a white solid. Analytical HPLC for **12e**: t_R = 15.5 min (10 to 50% solvent B over 30min, Agilent C18 column); ESI-MS: calcd for $C_{56}H_{100}N_{18}O_{18}$: 1313.5 Da (average isotopes), (*m/z*) $[M+H]^+$: 1313.8, $[M+2H]^{2+}$: 657.4; found: $[M+H]^+$: 1313.8, $[M+2H]^{2+}$: 657.5.

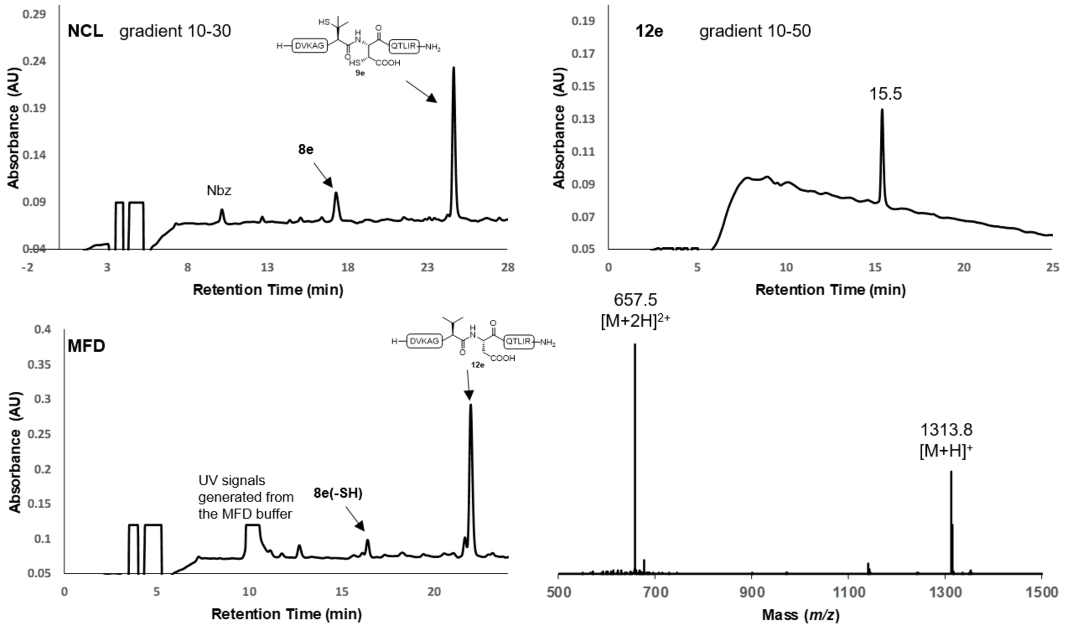

Figure S32. Top left: UV trace of ligation reaction between peptides **7a** and **8e**; Bottom left: UV trace of one-pot desulfurization following ligation; Top right: UV trace of purified **12e**; Bottom right: ESI-MS data of purified **12e**.

One-pot ligation and desulfurization reactions between 7a and 8f

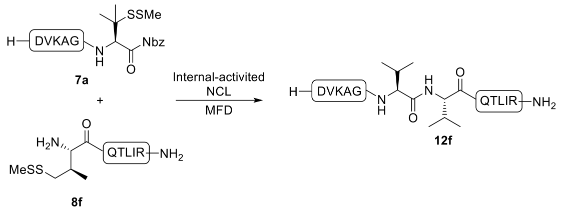

0.68 mg of peptidyl Nbz **7a** and 0.9 mg of cysteinyl peptide **8f** were subjected to the ligation conditions following General Procedure **3.5** as described previously. The reaction was stirred for 2 h at room temperature, followed by the addition of Bond-breaker® TCEP solution, ^{t}BuSH and VA-044 following General Procedure **3.7**. The reaction was stirred for another 3 h at 37 °C under an argon atmosphere, and quenched with 1.0 mL of $CH_3CN/H_2O/AcOH$ (5/90/5) solution. The resulting mixture was subjected to LC-MS analysis, and purified using preparative HPLC (10-30% solvent B over 30 min, Agilent Eclipse XDB-C18 column). The fractions containing pure peptide was concentrated affording **12f** (0.36 mg, 34%) as a white solid. Analytical HPLC for **12f**: t_R = 19.5 min (10 to 40% solvent B over 30min, Agilent C18 column); ESI-MS: calcd for $C_{57}H_{104}N_{18}O_{16}$: 1297.6 Da (average isotopes), (*m/z*) $[M+H]^+$: 1297.8, $[M+2H]^{2+}$: 649.4; found: $[M+H]^+$: 1297.7, $[M+2H]^{2+}$: 649.7.

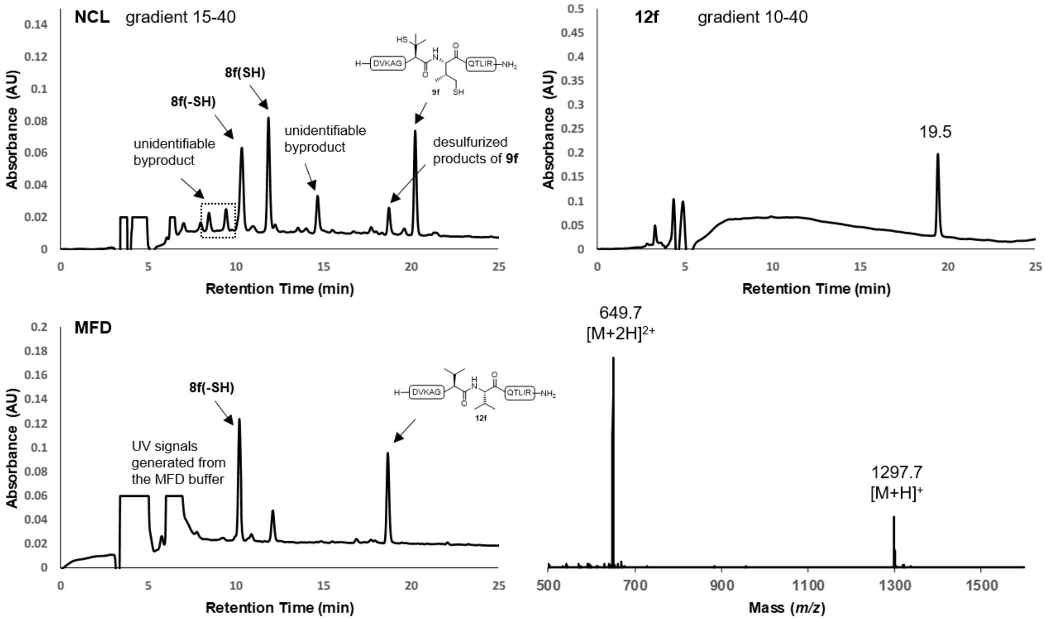

Figure S33. Top left: UV trace of ligation reaction between peptides **7a** and **8f**; Bottom left: UV trace of one-pot desulfurization following ligation; Top right: UV trace of purified **12f**; Bottom right: ESI-MS data of purified **12f**.

One-pot ligation and desulfurization reactions between 7a and 8g

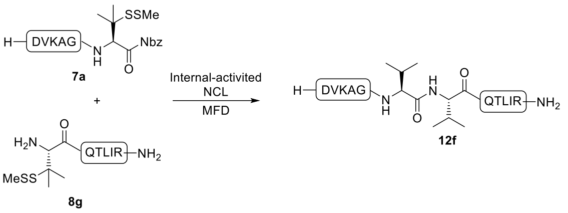

0.50 mg of peptidyl Nbz **7a** and 0.48 mg of cysteinyl peptide **8g** were subjected to the ligation conditions following General Procedure **3.5** as described previously. The reaction was stirred for 2 h at room temperature, followed by the addition of Bond-breaker® TCEP solution, *ᵗ*BuSH and VA-044 following General Procedure **3.7**. The reaction was stirred for another 3 h at 37 °C under an argon atmosphere, and quenched with 1.0 mL of $CH_3CN/H_2O/AcOH$ (5/90/5) solution. The resulting mixture was subjected to LC-MS analysis, and purified using preparative HPLC (10-30% solvent B over 30 min, Agilent Eclipse XDB-C18 column). The fractions containing pure peptide was concentrated affording **12f** (0.14 mg, 18%) as a white solid.

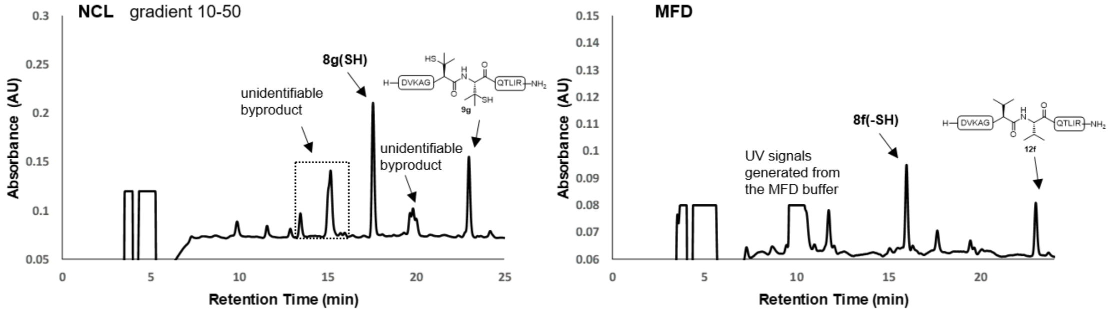

Figure S34. Left: UV trace of ligation reaction between peptides **7a** and **8g**; Right: UV trace of one-pot desulfurization following ligation.

One-pot ligation and desulfurization reactions between 7d and S9

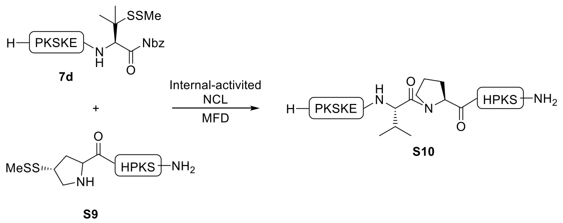

0.83 mg of peptidyl Nbz **7d** and 0.70 mg of cysteinyl peptide **S9** were subjected to the ligation conditions following General Procedure **3.5** as described previously. The reaction was stirred for 8 h at room temperature, followed by the addition of Bond-breaker® TCEP solution, *ᵗ*BuSH and VA-044 following General Procedure **3.7**. The

reaction was stirred for another 3 h at 37 °C under an argon atmosphere, and quenched with 1.0 mL of $CH_3CN/H_2O/AcOH$ (5/90/5) solution. As shown in Figure S35, only trace amount of ligation and desulfurized products were observed, which failed to afford isolable amount of product after preparative HPLC purification.

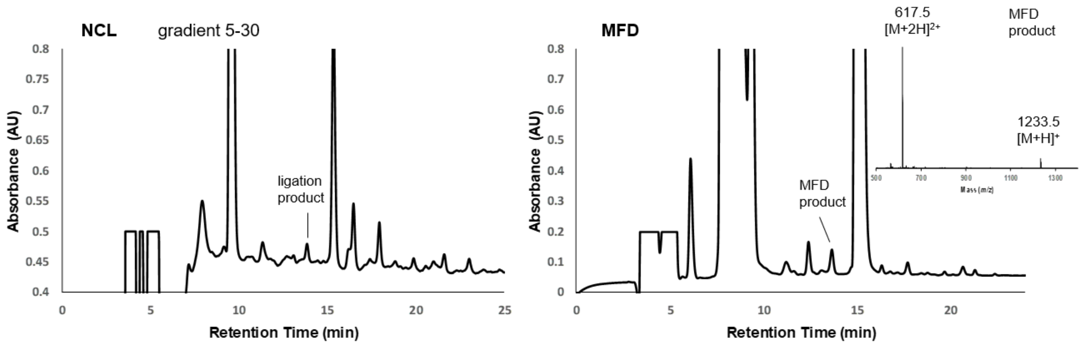

Figure S35. Left: UV trace of ligation reaction between peptides **7d** and **S9**; Right: UV trace of one-pot desulfurization following ligation.

One-pot ligation and desulfurization reactions between 7h and 8h

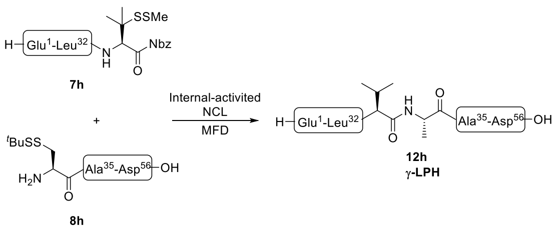

3.9 mg of peptidyl Nbz **7h** (1.0 equiv)and 4.7 mg of cysteinyl peptide **8h** (1.5 equiv) were subjected to the ligation buffer (6 M Gn·HCl, 200 mM Na_2HPO_4, 50 mM TCEP·HCl, pH 6.9) under an argon atmosphere, the concentration of the **7h** is approximately 5 mM, and the resulting solution was stirred at room temperature for 8 hours, followed by the addition of Bond-breaker® TCEP solution, ^{t}BuSH and VA-044 following General Procedure **3.7**. The reaction was stirred for another 8 h at 37 °C under an argon atmosphere, and quenched with 1.0 mL of $CH_3CN/H_2O/AcOH$ (5/90/5) solution. The resulting mixture was subjected to LC-MS analysis, and purified using preparative HPLC (15-30% solvent B over 30 min, Beim Brueckle C4 column). The fractions containing pure peptide was concentrated affording **12h** (3.5 mg, 55%) as a white solid. Analytical HPLC for **12h**: t_R = 24.7 min (15 to 35% solvent B over 30min, Beim Brueckle C4 column); ESI-MS: calcd for $C_{260}H_{400}N_{78}O_{89}S$: 6073.9 Da (average isotopes), (*m/z*) $[M+4H]^{4+}$: 1519.2, $[M+5H]^{5+}$: 1215.6, $[M+6H]^{6+}$: 1013.2, $[M+7H]^{7+}$:

868.6; found: $[M+4H]^{4+}$: 1519.3, $[M+5H]^{5+}$: 1215.7, $[M+6H]^{6+}$: 1013.2, $[M+7H]^{7+}$: 868.7.

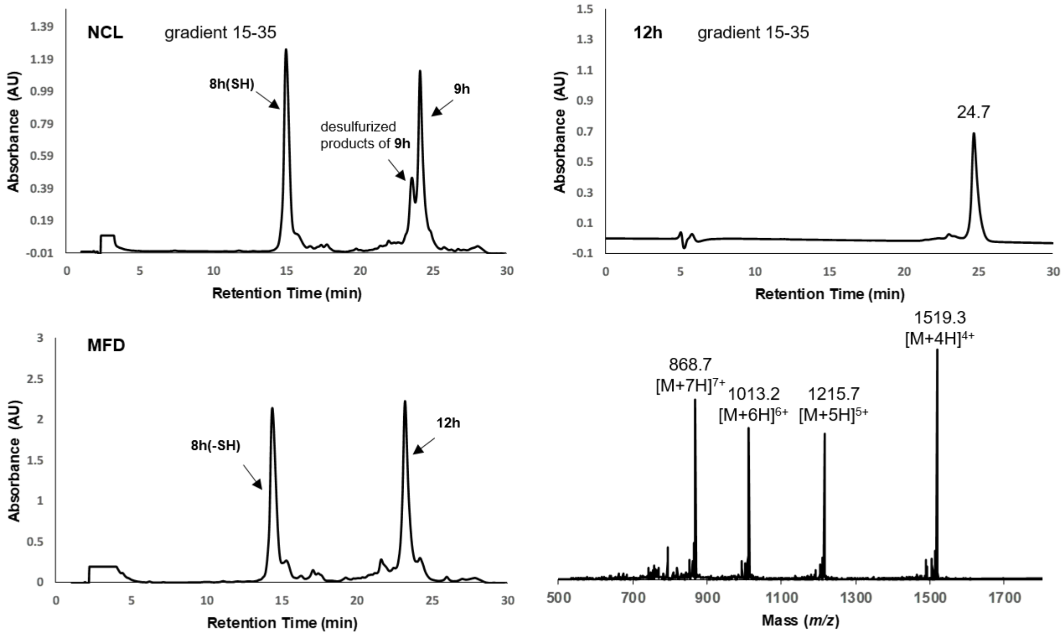

Figure S36. Top left: UV trace of ligation reaction between peptides **7h** and **8h**; Bottom left: UV trace of one-pot desulfurization following ligation; Top right: UV trace of purified **12h**; Bottom right: ESI-MS data of purified **12h**.

VII. Synthesis of [Nle^{45,63}]β-LPH (17) and [Nle^{8,18}]hPTH (23)

One-pot three segment ligation for the preparation of peptide 16

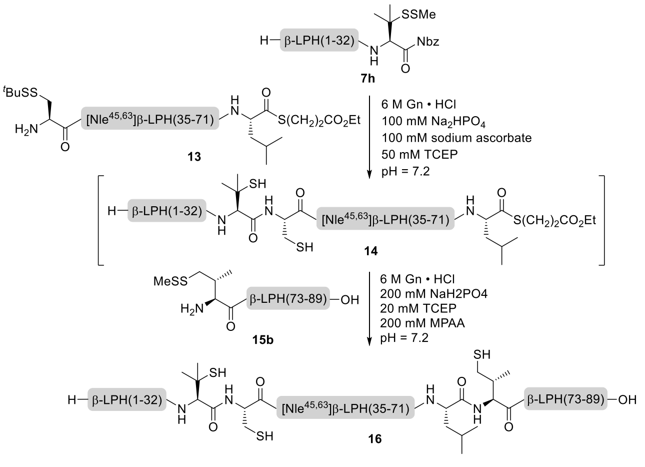

Peptidyl Pen-Nbz **7h** (2.6 mg, 1.0 equiv) and peptidyl thioester **13** (4.0 mg, 1.2 equiv) were subjected to the ligation buffer (6 M Gn·HCl, 100 mM Na_2HPO_4, 100 mM sodium ascorbate, 50 mM TCEP·HCl, pH 7.5) under an argon atmosphere, the concentration of the **7h** is approximately 5 mM, and the resulting solution was stirred for 8 h at room temperature. To the reaction mixture was then added a solution of **15b** (2.2 mg, 1.5 equiv) in buffer containing MPAA. The reaction was stirred for another 8 hours at room temperature, and quenched with 1.0 mL of CH_3CN/H_2O/AcOH (5/90/5) solution. The resulting mixture was subjected to LC-MS analysis, and purified using preparative HPLC (25-33% solvent B over 30 min, Proto-300 C4 column). The fractions containing pure peptide was concentrated affording **16** as a white solid. Analytical HPLC for **16**: t_R = 25.6 min (20 to 40% solvent B over 30min, Proto-300 C4 column); ESI-MS: calcd for $C_{432}H_{677}N_{123}O_{136}S_3$: 9866.1 Da (average isotopes), (*m/z*) $[M+5H]^{5+}$: 1974.0, $[M+6H]^{6+}$: 1645.2, $[M+7H]^{7+}$: 1410.3, $[M+8H]^{8+}$: 1234.1, $[M+9H]^{9+}$: 1097.1, $[M+10H]^{10+}$: 987.5, $[M+11H]^{11+}$: 897.8, $[M+12H]^{12+}$: 823.1; found: $[M+5H]^{5+}$: 1974.0, $[M+6H]^{6+}$: 1645.2, $[M+7H]^{7+}$: 1410.4, $[M+8H]^{8+}$: 1234.1, $[M+9H]^{9+}$: 1096.9, $[M+10H]^{10+}$: 987.5, $[M+11H]^{11+}$: 897.9, $[M+12H]^{12+}$: 823.1

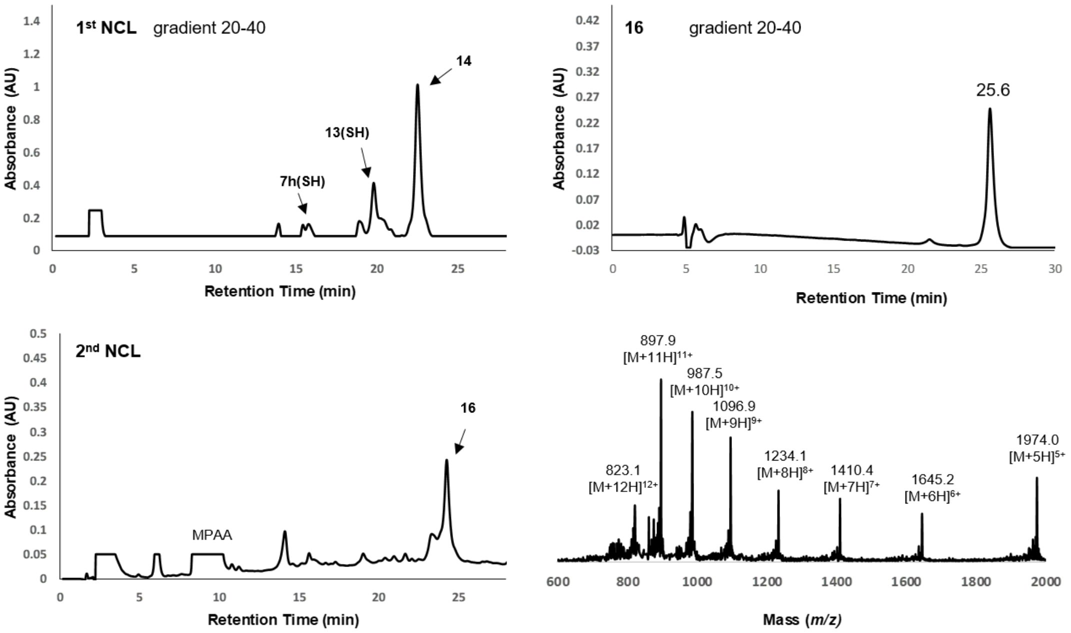

Figure S37. Top left: UV trace of ligation reaction between peptides **7h** and **13**; Bottom left: UV trace of one-pot ligation between **14** and **15**; Top right: UV trace of purified **16**; Bottom right: ESI-MS data of purified **16**.

Desulfurization of 16

Purified peptide **16** was subjected to desulfurization conditions following General Procedure **3.6** as described previously. The reaction mixture was allowed to stirred at 37 °C for 8 hours, and quenched with 1.0 mL of $CH_3CN/H_2O/AcOH$ (5/90/5) solution. The resulting mixture was subjected to LC-MS analysis, and purified using preparative HPLC (25-33% solvent B over 30 min, Proto 300-C4 column). The fractions containing pure peptide was concentrated affording **17** (2.2 mg, 31%, 3 steps) as a white solid. Analytical HPLC for **17**: t_R = 25.6 min (20 to 40% solvent B over 30min, Proto-300 C4 column); ESI-MS: calcd for $C_{432}H_{677}N_{123}O_{136}$: 9769.9 Da (average isotopes), (*m/z*) $[M+5H]^{5+}$: 1954.8, $[M+6H]^{6+}$: 1629.2, $[M+7H]^{7+}$: 1396.6, $[M+8H]^{8+}$: 1222.1, $[M+9H]^{9+}$: 1086.5, $[M+10H]^{10+}$: 977.9, $[M+11H]^{11+}$: 889.1, $[M+12H]^{12+}$: 815.1; found: $[M+5H]^{5+}$: 1954.8, $[M+6H]^{6+}$: 1629,3, $[M+7H]^{7+}$: 1396.7, $[M+8H]^{8+}$: 1222.0, $[M+9H]^{9+}$: 1086.2, $[M+10H]^{10+}$: 978.0, $[M+11H]^{11+}$: 889.1, $[M+12H]^{12+}$: 815.0.

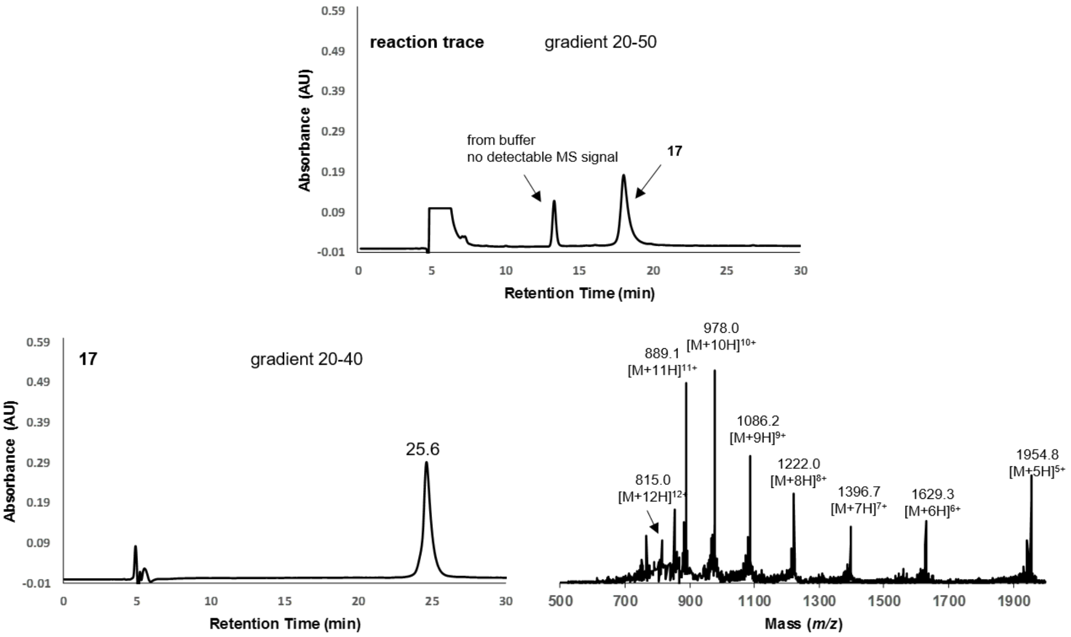

Figure S38. Top: UV trace of desulfurization of **16**; Bottom left: UV trace of purified **17**; Bottom right: ESI-MS data of purified **17**.

One-pot three segment ligation for the preparation of peptide 22

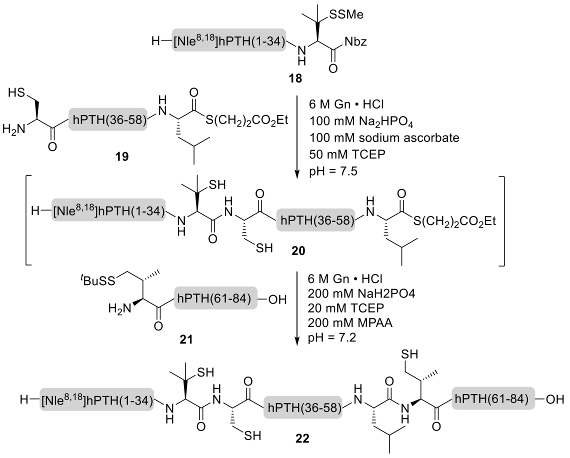

Peptidyl Pen-Nbz **18** (6.8 mg, 1.0 equiv) and peptidyl thioester **19** (5.0 mg, 1.2 equiv) were subjected to the ligation buffer (6 M Gn·HCl, 100 mM Na_2HPO_4, 100 mM sodium ascorbate, 50 mM TCEP·HCl, pH 7.5) under an argon atmosphere, and the resulting solution was stirred overnight at room temperature. To the reaction mixture was then added a solution of **21** (5.9 mg, 1.4 equiv) in buffer containing MPAA. The reaction was stirred for another 8 hours at room temperature, and quenched with 1.0 mL of $CH_3CN/H_2O/AcOH$ (5/90/5) solution. The resulting mixture was subjected to LC-MS analysis, and purified using preparative HPLC (27-38% solvent B over 30 min, Proto-300 C4 column). The fractions containing pure peptide was concentrated affording **22**

as a white solid with small amount of inseparable byproduct that could be removed in the next step. Analytical HPLC for **22**: t_R = 23.7 min (27 to 38% solvent B over 30min, Proto-300 C4 column); ESI-MS: calcd for $C_{410}H_{678}N_{126}O_{126}S_3$: 9484.9 Da (average isotopes), (*m/z*) $[M+5H]^{5+}$: 1897.6, $[M+6H]^{6+}$: 1581.5, $[M+7H]^{7+}$: 1355.7, $[M+8H]^{8+}$: 1186.4, $[M+9H]^{9+}$: 1054.7, $[M+10H]^{10+}$: 949.3, $[M+11H]^{11+}$: 863.1, $[M+12H]^{12+}$: 791.3, $[M+13H]^{13+}$: 730.5; found: $[M+5H]^{5+}$: 1898.0, $[M+6H]^{6+}$: 1581.8, $[M+7H]^{7+}$: 1365.1, $[M+8H]^{8+}$: 1186.8, $[M+9H]^{9+}$: 1054.9, $[M+10H]^{10+}$: 949.5, $[M+11H]^{11+}$: 863.3, $[M+12H]^{12+}$: 791.5, $[M+13H]^{13+}$: 730.7.

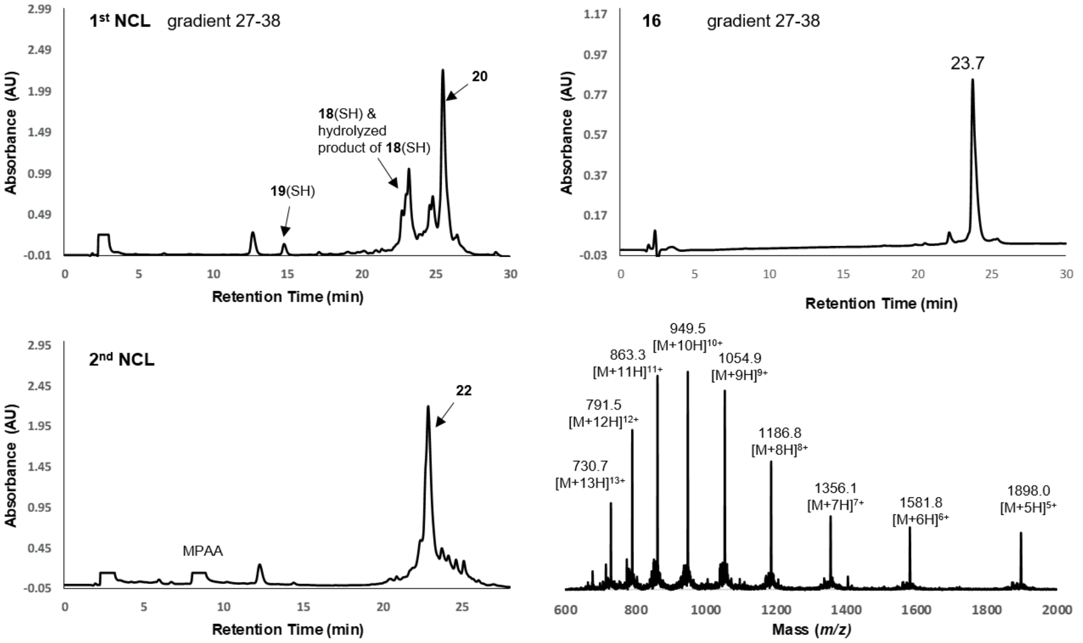

Figure S39. Top left: UV trace of ligation reaction between peptides **18** and **19**; Bottom left: UV trace of one-pot ligation between **20** and **21**; Top right: UV trace of purified **22**; Bottom right: ESI-MS data of purified **22**.

Desulfurization of 22

Peptide **22** was subjected to desulfurization conditions following General Procedure **3.6** as described previously. The reaction mixture was allowed to stirred at 37 °C for 8 hours, and quenched with 1.0 mL of $CH_3CN/H_2O/AcOH$ (5/90/5) solution. The resulting mixture was subjected to LC-MS analysis, and purified using preparative HPLC (27-38% solvent B over 30 min, Proto 300-C4 column). The fractions containing pure peptide was concentrated affording **23** (5.0 mg, 35%, 3 steps) as a white solid. Analytical HPLC for **23**: t_R = 22.4 min (20 to 40% solvent B over 30min, Proto 300-

C4 column); ESI-MS: calcd for $C_{410}H_{678}N_{126}O_{126}$: 9388.7 Da (average isotopes), (*m/z*) $[M+5H]^{5+}$: 1878.4, $[M+6H]^{6+}$: 1565.5, $[M+7H]^{7+}$: 1342.0, $[M+8H]^{8+}$: 1174.4, $[M+9H]^{9+}$: 1044.9, $[M+10H]^{10+}$: 939.7, $[M+11H]^{11+}$: 854.4, $[M+12H]^{12+}$: 783.3, $[M+13H]^{13+}$: 723.1; found: $[M+5H]^{5+}$: 1879.1, $[M+6H]^{6+}$: 1566.0, $[M+7H]^{7+}$: 1342.3, $[M+8H]^{8+}$: 1174.7, $[M+9H]^{9+}$: 1044.4, $[M+10H]^{10+}$: 940.0, $[M+11H]^{11+}$: 854.6, $[M+12H]^{12+}$: 783.5, $[M+13H]^{13+}$: 723.3.

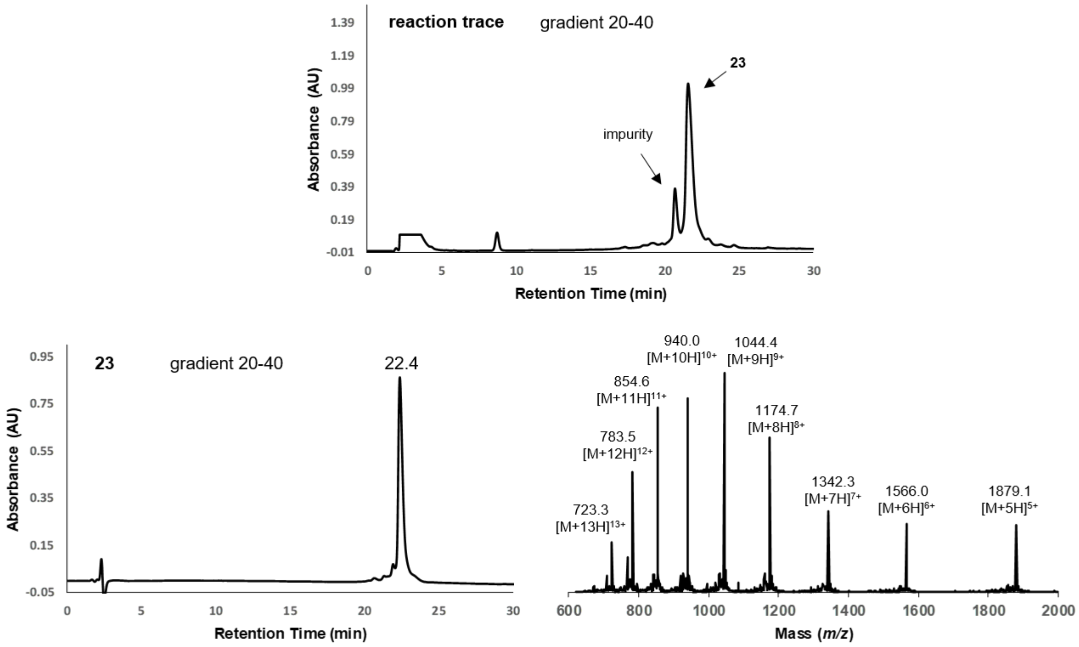

Figure S40. Top: UV trace of desulfurization of **22**; Bottom left: UV trace of purified **23**; Bottom right: ESI-MS data of purified **23**.
